# Profiling the nasopharyngeal Microbiome in patients with community-acquired pneumonia caused by *Streptococcus pneumoniae*: diagnostic challenges and ecological insights

**DOI:** 10.1007/s00430-025-00828-0

**Published:** 2025-04-10

**Authors:** Cristina Zubiria-Barrera, Linda Yamba Yamba, Tilman E. Klassert, Malena Bos, Jonas Ahl, Lisa Wasserstrom, Hortense Slevogt, Kristian Riesbeck

**Affiliations:** 1 Department of Respiratory Medicine and Infectious Diseases, MHH, German Center for Lung Research (DZL), BREATH, Hannover, Germany; 2 Respiratory Infection Dynamics Group, Helmholtz Centre for Infection Research, Braunschweig, Germany; 3 Cluster of Excellence RESIST (EXC 2155), Hannover Medical School, Carl-Neuberg-Straße 1, 30625 Hannover, Germany; 4 Clinical Microbiology, Department of Translational Medicine, Faculty of Medicine, Lund University, Malmö, Sweden; 5 Infectious Diseases, Department of Translational Medicine, Faculty of Medicine, Lund University, Malmö, Sweden; 6 Clinical Microbiology, Infection Control and Prevention, Skåne University Hospital, Lund, Sweden

**Keywords:** Community acquired pneumonia (CAP), Microbiome, Microbial interactions, Nasopharynx, Resilience, *Streptococcus pneumoniae*, Viral infection

## Abstract

Community-acquired pneumonia (CAP) is a significant health threat for adults. Although conjugate vaccines have reduced pneumococcal CAP incidence in children, *Streptococcus pneumoniae*-related CAP remains prevalent among older adults. The nasopharynx acts as a reservoir for *S. pneumoniae*, yet the interplay between this pathogen and the nasopharyngeal microbiome during and after pneumonia remains poorly understood. This study included 61 adult patients diagnosed with pneumococcal CAP and 61 matched healthy controls. An *S. pneumoniae*-specific PCR, urine antigen tests and bacterial cultures were performed. Nasopharyngeal swabs collected at admission and three months post-infection were analyzed for microbiome dynamics through *16 S rRNA* gene amplicon sequencing. *16 S rRNA* gene amplicon sequencing revealed *Streptococcus* spp. in the majority of all nasopharyngeal samples during infection compared to the other diagnostic test performed. While overall bacterial biomass did not differ between groups, patients exhibited higher alpha diversity (*p =* 0.012) and lower microbiome stability post-infection. Beta diversity analysis distinguished infection from healthy status (*p =* 0.002). Taxonomic analysis showed similar core microbiota across groups, but *Streptococcus* spp. was significantly more abundant during infection, particularly in those patients with viral co-infections. Notably, unique significant bacterial interactions were identified both during and after infection, as well as in healthy states. A negative correlation was observed between *Corynebacterium* and *Streptococcus* spp. in infected patients, suggesting a potential antagonistic interaction between these taxa. The nasopharyngeal microbiome in patients with pneumococcal CAP demonstrates persistent disruption post-infection, characterized by lower resilience three months after acute illness. Additionally, we identified specific bacterial interplays during and after infection that differed from those in healthy donors. These bacterial dynamics might play critical roles in pathogen colonization resistance and infection prevention. Thus, our findings highlight the need for further investigation into microbial interactions and potential microbiome-based therapies for respiratory infections, particularly in vulnerable populations.

**Supplementary Information:**

The online version contains supplementary material available at 10.1007/s00430-025-00828-0.

## Introduction

Community-acquired pneumonia (CAP) remains a critical public health issue, with *Streptococcus pneumoniae* being the most common causative pathogen [1]. Pneumococcal infections range from mild to severe, typically starting when the bacteria are inhaled into the lower airways, spreading through the respiratory tract, and causing pulmonary lobar pneumonia [2]. Individuals with conditions such as chronic obstructive pulmonary disease (COPD), coronary artery disease (CAD), or acute viral respiratory infections have an increased risk of developing severe pneumococcal pneumonia. These conditions impair immune function and lung capacity, facilitating bacterial invasion and leading to more severe disease [3]. Viral infections further weaken lung defenses, increasing susceptibility to secondary infections [4]. In such cases, the infection may spread and cause invasive disease with the risk of sepsis [3]. *Streptococcus pneumoniae* is a pathobiont, it is part of the normal host microbiome but can become pathogenic under certain circumstances. It frequently colonizes the nasopharynx of healthy individuals, typically without causing disease, and remains in an asymptomatic state [5]. Colonization of *S. pneumoniae* in the nasopharynx varies widely, with carriage rates ranging from 5 to 10% in healthy adults to 20–40% in children [2]. Thus, nasopharyngeal colonization of *S. pneumoniae* serves as a reservoir for infection, particularly in children and older adults, who face an increased risk due to age-related immune decline and comorbid conditions [6]. The introduction of pneumococcal conjugate vaccines (PCVs) in pediatric immunization programs has significantly reduced the incidence of pneumococcal CAP in children [7]. However, pneumococcal CAP with PCV related serotypes continues to be prevalent in the adult population [8]. It is also important to note that serotypes are correlated to the invasive disease potential of *S. pneumoniae*, where serotypes such as 3, 8 and 12 F are overrepresented in severe disease compared to their prevalence in carriage [9].

The nasopharynx harbors a dynamic microbial community essential for respiratory health and homeostasis [10, 11]. Research has shown that the composition of the nasal microbiome is also linked to *S. pneumoniae* acquisition and bacterial density, particularly during viral co-infections [12]. Baseline nasal microbiota composition significantly influences *S. pneumoniae* carriage outcomes, especially following viral infection. Even without direct colonization, exposure to *S. pneumoniae* may disrupt the nasal microbiota and affect host immune responses [12]. Importantly, the nasopharyngeal microbiome includes commensal bacteria that protect against pathogen colonization through direct mechanisms, such as competitive exclusion and the production of inhibitory substances, as well as indirect mechanisms, like enhancing host immune responses. Key protective commensals include *Corynebacterium* spp. and *Dolosigranulum* spp [13].

During the first year of life, the nasopharyngeal microbiome is predominantly colonized by genera such as *Moraxella*, *Streptococcus*, *Corynebacterium*, *Staphylococcus*, *Haemophilus*, and *Dolosigranulum* spp. However, the composition of this early microbiome shifts rapidly in response to environmental factors [10]. In adults, a healthy nasopharyngeal microbiome is typically diverse, with commensal bacteria playing a critical role in preventing pathogen overgrowth through colonization resistance [14]. These commensal-pathogen interactions regulate the microbial community, either inhibiting or facilitating pathogen colonization [15]. The development of a stable, healthy microbiome is shaped by numerous factors, including environmental conditions (e.g., temperature, humidity, and nutrient availability) and individual characteristics (e.g., age and genetics) [11]. Disruptions to this balance can lead to pathogen overgrowth, weakened infection resistance, and an increased risk of disease [15]. However, the extent to which microbiota composition contributes to pneumococcal colonization risk remains largely unexplored.

This study aims to investigate the nasopharyngeal microbiome in a pilot cohort of CAP patients with the detection of *S. pneumoniae* to better understand its role in pneumococcal pneumonia. We compared the nasopharyngeal microbiome of individuals infected with *S. pneumoniae* to that of age, sex and season-matched healthy controls, focusing on microbial composition, colonization dynamics, viral co-infections, and key clinical parameters. Additionally, we assessed how antibiotic treatment impacts the nasopharyngeal microbiome three months after discharge, exploring whether the microbial community stabilizes to resemble that of healthy individuals. Associations between *S. pneumoniae* and commensal microbes during infection and recovery were examined. By clarifying these dynamics, we identified key microbial taxa that might influence *S. pneumoniae* colonization in the nasopharynx and explored their impact on disease pathogenesis.

## Materials and methods

### Subjects

The ECAPS (“Etiology of community-acquired pneumonia in Sweden”) cohort was established between September 2016 and September 2018 and includes 518 patients along with 493 seasonally matched asymptomatic controls from the Department of Orthopedics (Supplemental data, Material and Methods) [8, 16]. Subjects underwent comprehensive testing to establish the etiology of CAP including both PCR, urine antigen detection methods and culture of blood and respiratory tract samples. For comparison, urine antigen detection methods, nasopharyngeal cultures, and swabs for PCR of selected viruses and bacteria were also performed on most healthy controls (Supplemental data, Material and Methods) [8, 16].

Patients were selected for microbiome analysis based on the finding of *S. pneumoniae* using a nasopharyngeal PCR (*lytA*), urine antigen test (UAT) BinaxNOW *S. pneumoniae*^®^, serotype specific antigen detection in urine (UAD) or culture positivity in nasopharyngeal, blood, or lower respiratory tract samples. Subjects were sampled with a nasopharyngeal swab at inclusion for detection of viral/bacterial pathogens as well as microbiome analysis. A second nasopharyngeal swab was collected from patients 10–17 weeks after discharge for comparison. Patients (*n* = 61) were selected for microbiome analysis based on the finding of *S. pneumoniae* (Figure S1 and S2) [16]. In addition, the same number of age, sex- and season-matched healthy controls (*n* = 61) were included in the analyses. Ethical approval was granted by Lund Regional Ethics Committee (Nos 2016/220 and 2016/340), and written informed consent was obtained from all patients.

### DNA extraction and *16 S rRNA* gene quantification

DNA was extracted from nasopharyngeal swab samples and negative controls (Ultrapure DNAse/RNAse free water) using magLEAD12gC along with the magLEAD Consumable Kit (Precision System Science, Matsudo, Japan). Thereafter, a SYBR-Green-based quantitative PCR (qPCR) reaction (BioLine, UK) combining DNA template with specific amplification primers targeting the V4 region of the *16 S rRNA* gene was used. qPCR reactions were conducted in technical duplicates using a Rotor-Gene Q cycler (QIAGEN, the Netherlands). Absolute quantification of target copies was achieved using a *16 S rRNA* standard.

### *16 S rRNA* gene library construction and sequencing

Amplification of the *16 S rRNA* gene was performed with fused primers 515 F/806R, incorporating Golay barcodes and adapter sequences (Table S1). PCR reactions were run in an S1000 Thermal Cycler (BioRad). Resulting PCR products were purified by 2% SizeSelect E-Gels (Thermo Fisher Scientific, state) and quantified on D1000 Tapes using a TapeStation 2200 (Agilent Technologies, UK). Equimolar pooled purified samples were processed by the MiSeq Reagent Kit v2 (Illumina) and sequenced on a MiSeq apparatus (Illumina).

### *16 S rRNA* gene amplicon sequencing analysis

Fastq files underwent demultiplexing using the QIIME2 bioinformatic software (version 2022.11) [17]. Quality control of sequence data was performed using the DADA2 denoising pipeline [18]. A custom-developed contaminant feature-filtering bioinformatics pipeline was applied to the dataset to remove potential contaminants originating from negative controls. Eight negative controls samples were sequenced, each corresponding to a distinct DNA extraction procedure. This bioinformatics pipeline can be executed in the R software. A brief workflow of the bioinformatics process can be seen in the Supplemental Material (Figure S3). Briefly, the dataset was divided into subject and negative control samples. First, the mean relative abundance of each feature ID (FRA) was calculated for both groups. Feature IDs with a mean relative abundance (RA) of less than 1% in negative controls were discarded to prevent the misclassification of sequences as contaminants due to sequencing errors and/or index hopping. A ratio between the mean FRA of negative control samples and subject samples was then calculated. Features IDs with a ratio > 0.9 were classified as contaminants and removed from the subject dataset. The detailed pipeline is available on the public online repository GitHub (https://github.com/cbar123/Feature-filtering-pipeline-in-R.git). Taxonomy assignment of the filtered dataset was accomplished using a pre-trained Naive Bayes classifier on the SILVA database Ref NR 99 (v138) [19]. Differential abundance analysis of bacterial composition at genus level was conducted using the ‘ancombc’ R software package (v. 2.3.1). For diversity analysis of the microbiome data, the ‘phyloseq’ R package (v. 1.45.0) was used. To evaluate taxa richness (observed taxa) and evenness (abundance distribution) within samples, we calculated Observed OTUs, Shannon, and Simpson indices as measures of alpha diversity. For beta diversity comparisons, Bray-Curtis distances were used to generate non-metric multidimensional scaling (NMDS) plots, visualizing microbiome diversity differences between sample groups. The datasets (fastq files) generated are accessible in the SRA online repository under the Bioproject number: PRJNA1172557.

### Correlation analysis and statistics

The Fisher’s exact test and the Mann-Whitney U test in R (version 4.3.0) were used for statistical analyses and multiple comparisons adjusted with the Bonferroni method, unless otherwise specified. Pearson correlation analyses between bacterial relative abundance and clinical data were performed using the ‘stats’ and ‘ggpubr’ R packages (version 4.3.0 and 0.6.0, respectively). Co-occurrence network analyses of bacterial taxa were conducted with the SparCC function of the ‘SpiecEasi’ R package (version 1.1.3) using 100 bootstrap iterations. Networks were visualized using the Gephi software (version 0.10.1). Multivariate homogeneity of group dispersion was analyzed with the ‘vegan’ R package (version 2.6.4) and its ‘betadispr’ function. Beta diversity metrics between groups were compared using the PERMANOVA statistical test. The threshold for statistical significance was set at *p* < 0.05.

## Results

### Characteristics of laboratory samples obtained from patients diagnosed with CAP

Our study included patients with pneumococcal CAP during the acute infection phase, with nasopharyngeal samples collected at the time of diagnosis and admission to the hospital. The same patients were sampled again three months post-infection. Matched asymptomatic control subjects were also analyzed for comparison. In total, 61 patients with CAP and 61 healthy controls were included (Table 1). COPD, CAD, and previous or current smoking were significantly more common among CAP patients compared to controls.

**Table 1:** Cohort description

	CAP patients	Healthy Controls	
*n* (%)	*n* = 61	*n* = 61	*p* ^b^
Age (Years), median [min, max]	69 [22, 96]	68 [25, 90]	0.94
Sex Female	31 (51)	31 (51)	1
Body mass index, median [min, max]	24.5 [15.8, 37.1]	25.1 [17.0, 39.7]	0.45
Smoker			
Yes	14 (23)	8 (13)	0.24
Previous Smoker	25 (41)	16 (26)	0.19
No	22 (36)	37 (61)	0.033
COPD	17 (28)	1 (1.6)	< 0.001
Asthma	6 (10)	5 (8.2)	1
Congestive heart failure	7 (11.5)	4 (6.6)	0.53
Coronary artery disease	16 (26)	5 (8.2)	0.015
Autoimmune disease	3 (5)	2 (3.3)	1
Diabetes	9 (15)	8 (13)	1
Liver disease	0 (0)	1 (1.6)	1
Gastroesophageal reflux disease	10 (16)	5 (8.2)	0.27
Cancer - solid tumor	12 (20)	7 (11.5)	0.32
Cancer - hematologic	0 (0)	1 (1.6)	1
Chronic kidney disease	2 (3.3)	2 (3.3)	1
Antibiotic 14 days prior to admission			
Yes	6 (9.8)	4 (6.6)	0.75
Unknown	0 (0)	10 (16.4)	
CRB65 score 3 to 4	1 (1.6)	-	
PSI score, median [IQR]	78 [61–105]	-	
PSI grade IV-V	24 (39)	-	
Length of stay, median [IQR]	5 [4–8]	-	
Readmission ^a^	4 (6.6)	-	

^**a**^ Four patients were readmitted during the follow-up period, 50–90 days before convalescent sampling of the individual, no data available on length of stay during second admission

^**b**^ Bold figures indicate statistically significant differences

For clinical diagnosis, specific PCR tests from nasopharyngeal samples for *S. pneumoniae* (targeting the *lytA* gene) and other potential pathogens, along with standard microbiological cultures (nasopharynx, lower respiratory tract and blood) and urine antigen tests, were performed (Table 2). Patients who tested positive for *S. pneumoniae* in any test were included in this study (**Table S2**). Samples from these patients were additionally analyzed using *16 S rRNA* (V4 region) amplicon sequencing for microbiome investigation. This analysis revealed *Streptococcus* spp. in all but one nasopharyngeal sample, with feature counts (FC) ranging from 54 to 154,588 (median 2,697). The specific PCR test for *S. pneumoniae* (*lytA*) was positive in 58% of the samples tested. Notably, all samples containing more than 10,000 FC of *Streptococcus* spp. were positive in *lytA* PCR. Additionally, pneumococci were detected in 16% of the nasopharyngeal cultures. Most patients (69%) tested positive for the pathogen using the UAD, while 44% were positive with the UAT. Blood cultures identified the bacterial species in 16% of the cases. However, despite all patients being diagnosed with *S. pneumoniae* infection, none of the tests were positive for all samples. Serotyping of available isolates (*n* = 45) revealed that the most common serotypes of included patients were serotype 3 (27%) followed by 8 (11%) and 9 N (11%) (Table S3).

**Table 2:** Microbiological diagnostic testing

	CAP^a^ patients	Healthy Controls
	*n*/*N* (%)^b^	*n*/*N* (%)
UAD	42/61 (69)	1/46 (2.2)
Pneumococcal UAT	27/61 (44)	1/46 (2.2)
Nasopharyngeal PCR ^c^		
*Streptococcus pneumoniae*	35/60 (58)	2/34 (6)
*Haemophilus influenzae*	4/60 (6.7)	0/34 (0)
*Mycoplasma pneumoniae*	1/60 (1.7)	0/34 (0)
Viruses detected	15/60 (25)	2/34 (6)
Rhino/Enterovirus	3 (5)	0
Influenza A H3N2/H1N1	2 (3.3)	0
Influenza B	2 (3.3)	1 (2.9)
Human metapneumovirus	2 (3.3)	0
RSV A/B	2 (3.3)	0
Coronavirus OC43, NL63, 229E	3 (5)	1 (2.9)
Parainfluenza virus 1–3	1 (1.7)	0
Nasopharyngeal swab before empirical antibiotics	1/60 (1.6)	-
Nasopharyngeal culture		
*Streptococcus pneumoniae*	8/49 (16)	0/61 (0)
*Haemophilus influenzae*	3/49 (6)	0/61 (0)
*Moraxella catarrhalis*	7/49 (14)	2/61 (3.3)
*Streptococcus agalactiae*	1/49 (2)	
Nasopharyngeal culture before empirical antibiotics	36/47 (77)	-
Blood culture		
*Streptococcus pneumoniae*	15/58 (26)	-
Sputum culture		
*Streptococcus pneumoniae*	1/4 (25)	-

Abbreviations; CAP; community acquired pneumonia; UAD– urinary antigen detection; UAT– urine antigen test

^a^ In addition to presented data one patient had a bronchial washing performed with the detection of Streptococcus pneumoniae, Streptococcus agalactiae, Escherichia coli, Pseudomonas aeruginosa and Staphylococcus aureus

^b^ n/N = Number of positive findings/Number of tested patients

^c^ No finding of additional bacteria (*Chlamydia pneumoniae*, *Bordetella parapertussis* or *Bordetella pertussis*) or viruses (Parechovirus and Adenovirus)

### Post-infection nasopharyngeal Microbiome showed reduced bacterial biomass, increased alpha diversity, and lower Microbiome stability compared to infection and control groups

**Fig. 1:**
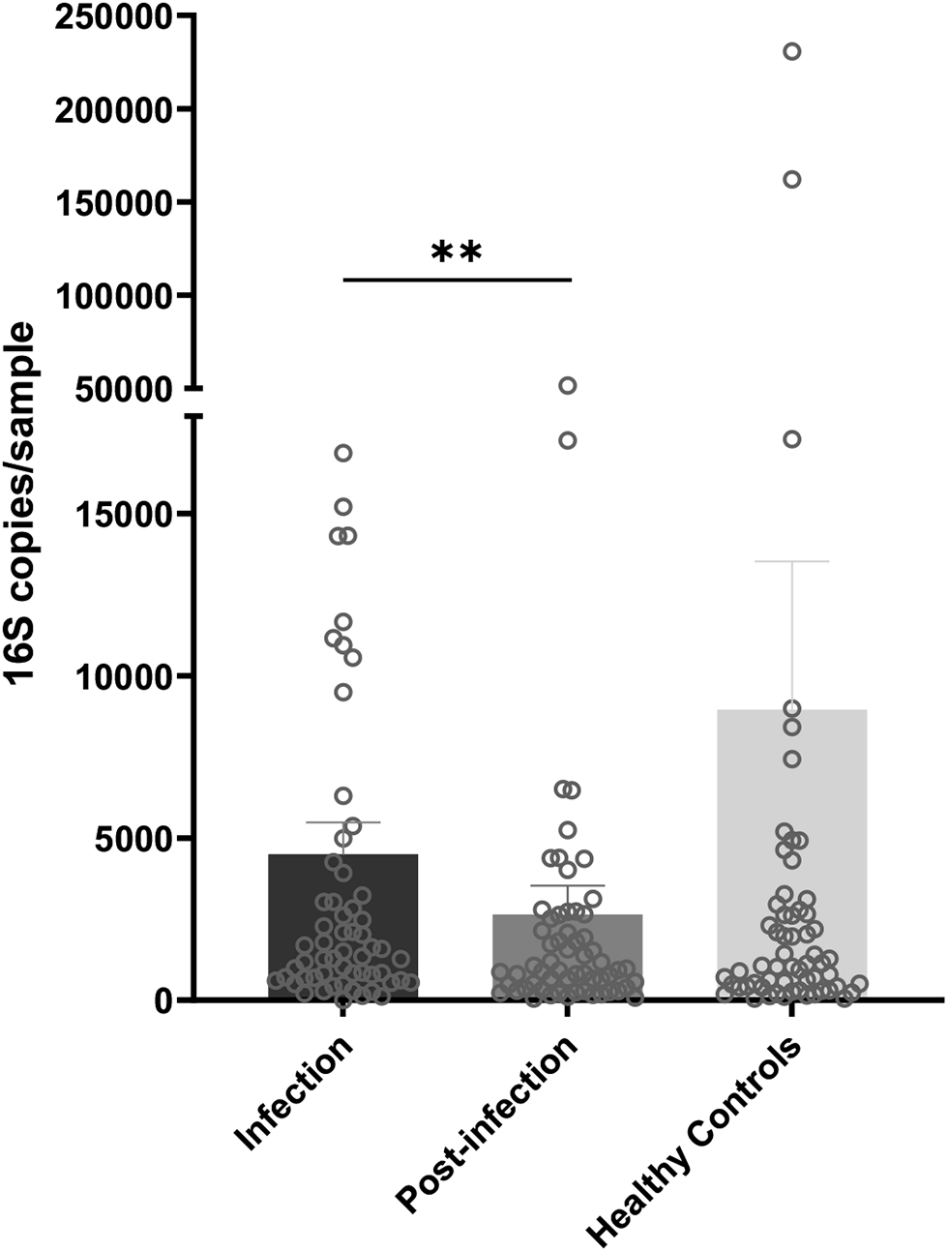
Bacterial biomass of nasopharyngeal swab samples. Scatter dot plot depict the quantitative analysis of bacterial biomass measured by qPCR, represented as *16 S rRNA* gene copies per sample. Statistical significance was assessed using pairwise comparisons with the Wilcoxon test, with *p*-values < 0.01 indicating significant differences

Nasopharyngeal samples from patients with *S. pneumoniae* CAP were compared with a matched control group of healthy volunteers. Additionally, patient samples collected at different time points were also included in the analyses. Absolute quantification of the *16 S rRNA* gene copies showed no statistical differences between the nasopharyngeal bacterial biomass of pneumococcal pneumonia patients and matched control samples (Fig. 1). In contrast, significant lower bacterial load was found in post-infection samples compared to those collected during infection (*p* = 0.004).

**Fig. 2:**
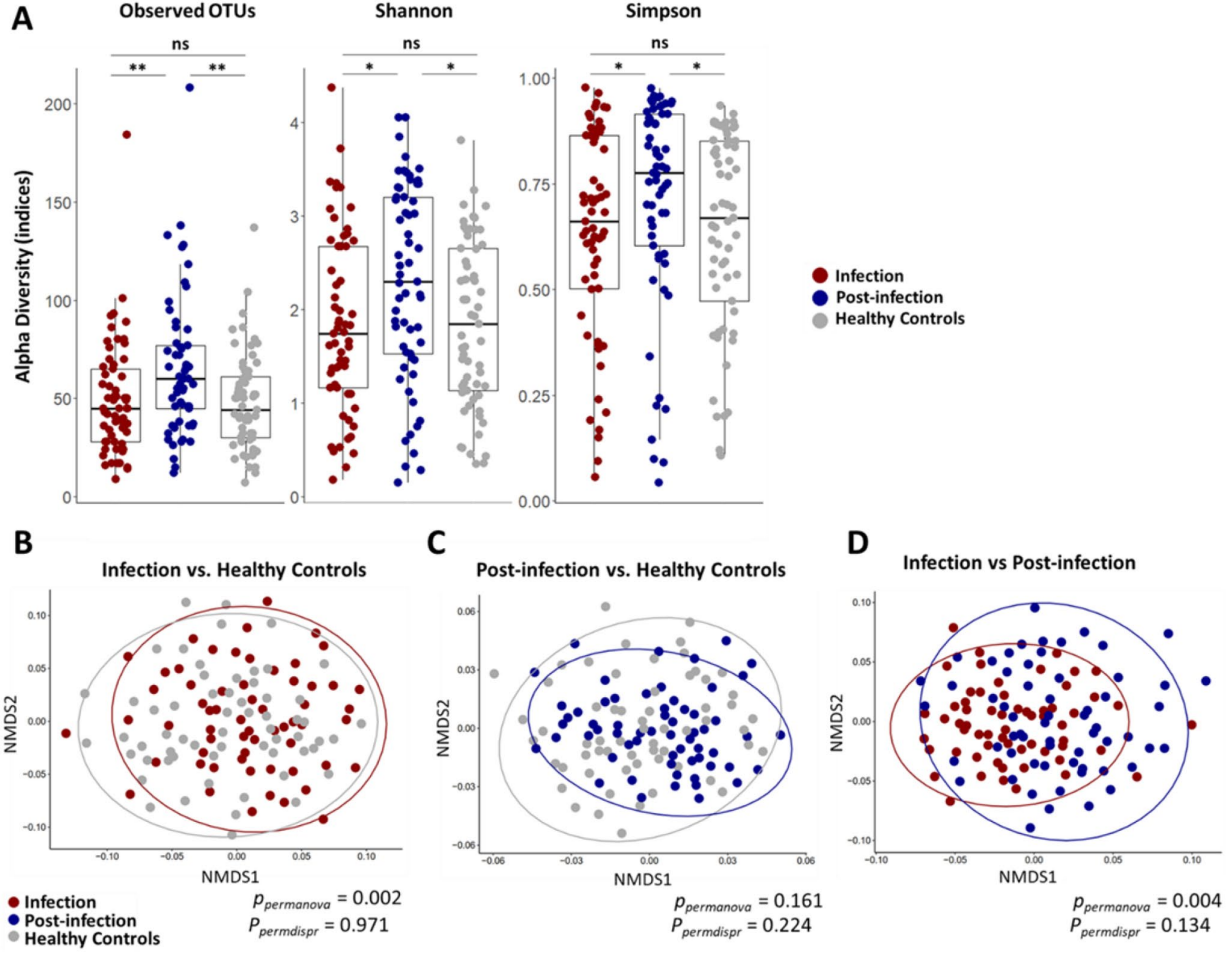
Diversity measurements between nasopharyngeal samples from *S. pneumoniae* CAP patients and healthy controls. **(A)** Box plots showing different indices for alpha diversity measurements of nasopharyngeal swab samples from *Streptococcus pneumoniae* CAP patients and healthy subjects. Comparisons are made between different sample collection time points and healthy controls. *P*-values from pairwise comparisons are derived using the Mann-Whitney U-test. **(B-C-D)** Bray Curtis distances were calculated to assess for the comparison of microbiome diversities between groups (beta diversity). Sample distances were plotted as non-metric multidimensional scaling (NMDS) plot. P-values from pairwise comparisons are determined using PERMANOVA and the permutation test for beta dispersion (betadisper). * indicates *p* < 0.05

To evaluate microbiome diversity within the samples, different metrics examining taxa richness and evenness were calculated. These included, Observed OTUs: measures the total number of taxa present in a sample, Shannon index: accounts for both richness and evenness, with a stronger emphasis on richness, and the Simpson index: similar to Shannon but places greater weight on evenness. The comparison between samples showed that nasopharyngeal samples from the infection phase and the control group showed no significant differences in the amount of taxa observed (richness) and abundances (evenness) measured (Observed OTUs *p* = 0.9, Shannon *p* = 0.91, Simpson *p* = 0.92) (Fig. 2A). In contrast, α-diversity indices differed significantly between infection-phase and post-infection samples (Observed OTUs: *p* = 0.0035, Shannon: *p* = 0.012, Simpson: *p* = 0.03). This indicates that post-infection samples had a higher number of species and a more even distribution of abundances compared to infected patients. Notably, the same results were observed when comparing post-infection samples with those from control individuals (Observed OTUs *p* = 0.0019, Shannon *p* = 0.011, Simpson *p* = 0.022). To assess for microbiome stability, we performed co-occurrence network analyses of significant bacterial correlations of each sample group and calculate the modularity index. This measurement indicates how well a network can be divided into distinct subgroups or modules, interpreting higher values (M = 0.4) as stable ecosystems [20]. Post-infection samples showed lower modularity (M = 0.296), as compared to patients infected samples (M = 0.341) suggesting lower microbial resilience. In contrast, the healthy microbiome exhibited the greatest stability (M = 0.429) (Figure S4).

Analysis of beta diversity, estimating for differences in microbiome diversities between groups, revealed significant differences between patient samples during infection and healthy controls (*p* = 0.002) (Fig. 2B). In contrast, post-infection samples showed similar beta diversity distances between these two groups (Fig. 2C). Moreover, Bray-Curtis distances indicated significant differences in microbiome diversity between samples collected during infection and post-infection phases (*p* = 0.004) (Fig. 2D).

### Taxonomic analysis revealed similar bacterial community structures between pneumonia patients and healthy controls, with notable interindividual variability and a significant decrease in *Streptococcus* spp. Abundance post-infection

Analysis of the taxonomic bacterial composition of nasopharyngeal swab samples from individuals and negative controls revealed similar taxonomic profiles and microbiome diversities (Figure S5 and S6). To improve accuracy of our microbiome analysis, we developed a custom bioinformatics pipeline that detects and eliminates contaminants found in negative controls from the dataset.

**Fig. 3:**
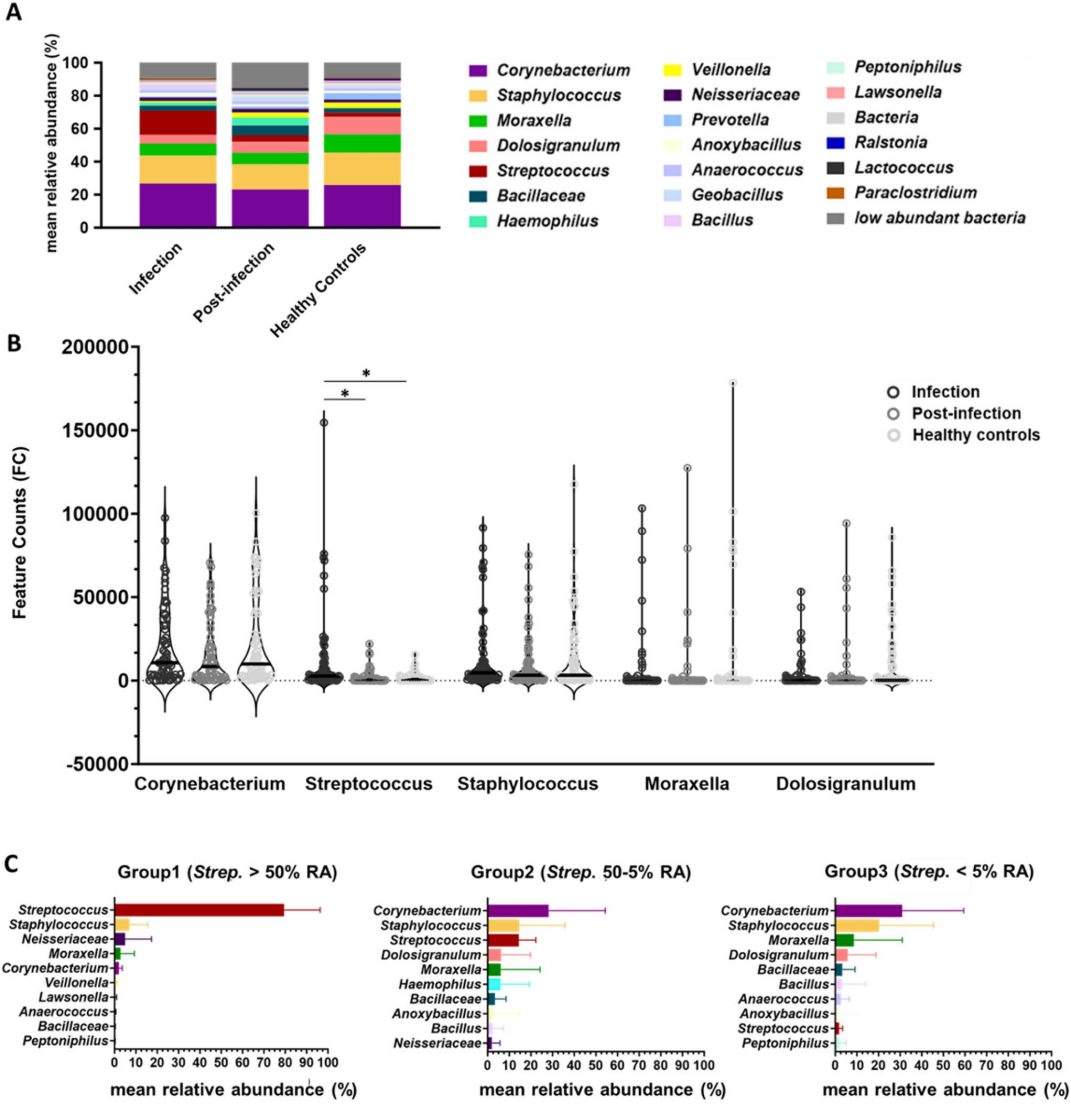
Taxonomic composition of nasopharyngeal bacterial communities in *S. pneumoniae* CAP patients and healthy controls. (**A**) Mean relative abundances (RA) of the 20 most abundant genera are illustrated for each group. (**B**) Violin plot showing the median RA of the five most representative bacterial genera across the three study groups, with ANCOMBC statistical significance indicated. (**C**) Mean relative abundance of the top 10 bacterial taxa in each pneumococcal CAP sample group, classified according to the abundance of *Streptococcus* spp. (*Strep*.): Group 1 (*Strep.* > 50% RA), Group 2 (*Strep.* 50%-5% RA), and Group 3 (*Strep.* <5% RA)

Bacterial taxonomic analysis of the filtered dataset revealed a high similarity in bacterial communities among samples from *S. pneumoniae* pneumonia patients during infection, post-infection, and healthy controls (Fig. 3A). The genera *Corynebacterium* spp., *Streptococcus* spp., *Staphylococcus* spp., *Moraxella* spp., and *Dolosigranulum* spp. were the dominant bacterial taxa in the nasopharyngeal microbiome across all analyzed samples (Table S4). Figure 3B illustrates the median abundance of these five genera across the groups. We observed high interindividual variation within groups, with samples exhibiting both high and low feature counts (FC) for the same bacterium. Notably, only the FC of *Streptococcus* spp. decreased progressively from the infection phase (median FC: 2,697) to post-infection (median FC: 375), compared to healthy individuals (median FC: 706).

Due to the high interindividual variation observed in bacterial communities across different groups (Figure S7A), we explored taxonomic differences among the entire sample set. An analysis of compositions of microbiomes with bias correction (ANCOMBC) was performed. This statistical test revealed a significantly increased abundance of *Streptococcus* spp. in patient samples during infection compared to post-infection and healthy nasopharyngeal samples, among other findings (Table S5). Since amplicon sequencing of the V4 region cannot precisely identify bacterial species, we used clinical diagnostic data to correlate detected *Streptococcus* spp. with diagnosed *Streptococcus pneumoniae*. Pearson correlation analysis found a significant association between the relative abundance of *Streptococcus* spp. and *S. pneumoniae* PCR test results in infected patient samples (Table S6), indicating that *Streptococcus* spp. detected through *16 S rRNA* amplicon sequencing in samples from patients during infection is likely to be *Streptococcus pneumoniae*.

### Distinct significant bacterial associations in the nasopharyngeal Microbiome of Pneumococcal CAP patients during infection and post-infection compared to healthy subjects

Given the high interindividual variance within this cohort but the significantly increased RA of *Streptococcus* spp. during infection, nasopharyngeal samples from pneumonia patients suffering from *S. pneumoniae* at this acute stage were categorized based on the RA of *Streptococcus* spp., likely *Streptococcus pneumoniae*. In Figure S7B, Group 1 included patients with > 50% RA. Group 2 contained samples with 50%-5% RA, and, finally, Group 3 patients with < 5% RA of *Streptococcus* spp. Figure 3C displays the mean RA of the 10 most abundant bacterial taxa in each group. Group 1 showed *Streptococcus* spp. as the dominant taxon (mean RA 79.5%) while other taxa, like *Corynebacterium* spp. showed to be less (mean RA 2.1%). In contrast, *Corynebacterium* spp. emerged as the predominant genus in Groups 2 and 3, with mean RAs of 28.2% and 30.9% respectively, while *Streptococcus* spp. showed decreased mean RA of 14.4% and 1.8%.

**Fig. 4:**
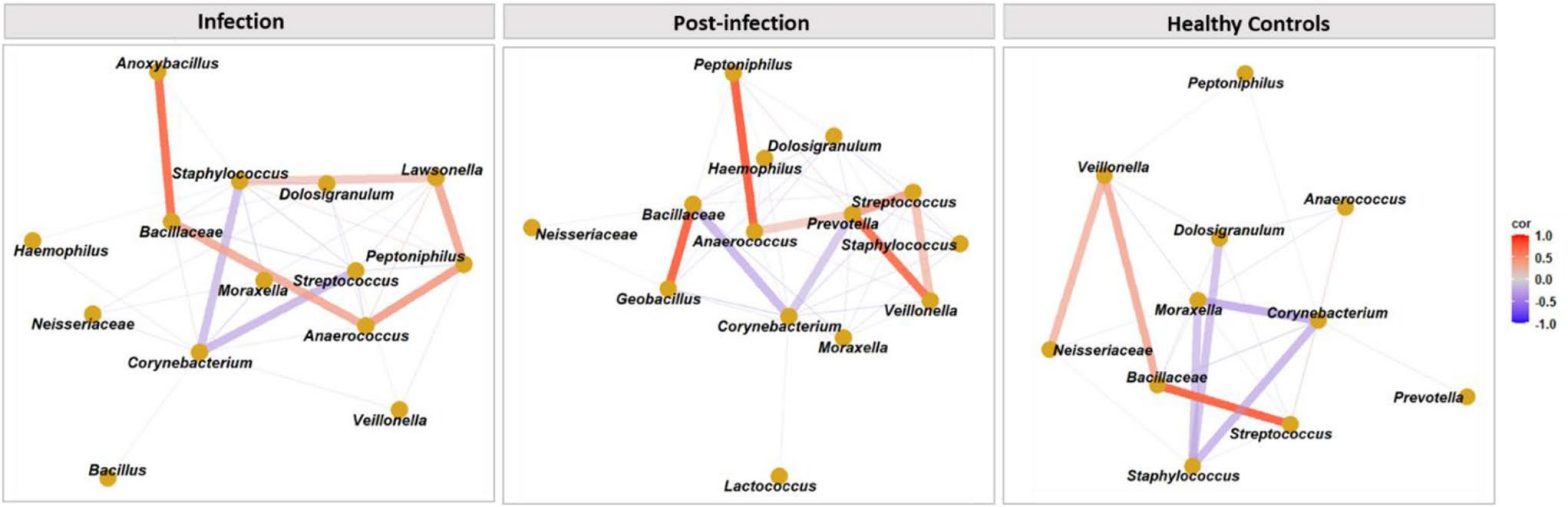
Network visualization of Pearson Correlation Coefficients (PCC) among bacterial taxa in nasopharyngeal samples. The network diagram illustrates Pearson correlation coefficients (PCC > 0.1) for bacterial taxa with a mean relative abundance greater than 1% in nasopharyngeal swab samples from both *S. pneumoniae* CAP patients and healthy controls. Positive PCCs are depicted in red, while negative PCCs are shown in blue. Significant positive and negative associations are represented by thick lines, while non-significant associations are shown with thin lines. A positive bacterial association suggests that both bacteria are likely to be present together in a sample, while a negative correlation indicates that if one bacterium is present, the other might be absent

To better understand the microbial interplay between *Streptococcus* spp. and other bacterial taxa during infection, post-infection, and healthy states, we selected for bacteria with a mean relative abundance (RA) of more than 1% and performed Pearson Correlation Coefficient (PCC) analysis (Table S7). To enhance data visualization, we also conducted a network analysis of significant bacterial associations (Fig. 4). During the infection phase, *Corynebacterium* spp. showed significant negative correlations with both *Streptococcus* and *Staphylococcus* spp. Interestingly, this antagonistic relationship between *Corynebacterium* spp. and *Staphylococcus* spp. was also observed in healthy samples. Additionally, in healthy individuals, significant negative correlations were found between *Corynebacterium* spp. and *Moraxella* spp., as well as between *Dolosigranulum* spp. and *Staphylococcus* spp. In the post-infection phase, significant positive correlations were observed between *Streptococcus* spp. and the bacterial taxa *Prevotella* spp. and *Veillonella* spp. indicating a shift in microbial dynamics post-infection. Conversely, *Corynebacterium* spp. was negatively associated with *Prevotella* spp.

*Staphylococcus* spp. exhibited several significant associations with other bacteria across the different phases. During the infection phase, it showed a significant positive correlation with *Lawsonella*, whereas in healthy controls, it had significant negative correlations with *Moraxella* and *Dolosigranulum*. On the other hand, *Bacillaceae* consistently showed significant positive correlations with other bacteria depending on the phase: during infection, it correlated positively with *Anaerococcus* and *Anoxybacillus*, whereas in healthy controls, it showed positive correlations with *Veillonella* and *Streptococcus* spp.

In the post-infection phase, *Corynebacterium* spp. developed significant negative correlations with *Prevotella* spp. and *Bacillaceae*. *Anaerococcus* continued to show significant positive correlations with *Prevotella* and *Peptoniphilus*. Notably, *Streptococcus* spp., which had significant negative correlations with *Corynebacterium* spp. during the infection phase, shifted to significant positive correlations with *Veillonella* and *Prevotella* spp. in the post-infection phase.

These findings reveal intriguing shifts in microbial interactions across infection, post-infection, and healthy states. To explore these bacterial interactions further, we conducted a focused literature search. Using keywords such as “bacterial association” and/or “bacterial correlation,” we reviewed relevant studies on bacterial interactions within the nasopharyngeal microbiome of adults with pneumococcal pneumonia up to August 2024. Our search included literature on interactions in the same or other respiratory sites, as well as other body areas, and examined both infection and healthy states, with a particular focus on adults and children (**Table S8**). Interestingly, many of the significant bacterial associations identified in the nasopharynx in this study have not been previously described. Similar associations have been reported in other parts of the body, such as the lungs, oral cavity, and skin, but not specifically in the nasopharynx. For example, positive associations between *Streptococcus* spp. and *Veillonella* spp. have been observed in the oral cavity, while positive correlations between *Streptococcus* and *Prevotella* spp. have been noted in the lungs. Another notable finding is the negative association between *Corynebacterium* and *Streptococcus* spp., which has been documented in the nasopharynx of healthy children but not in adults. Similarly, the negative association between *Corynebacterium* and *Moraxella* spp. has been observed in infants but not in adults.

### Correlation analysis revealed a significant association between *Streptococcus* spp. Abundance and viral co-infection in the nasopharynx of Pneumococcal pneumonia patients

To further investigate the presence of *Streptococcus* spp. in the nasopharyngeal microbiome of these patients, we performed Pearson correlation analysis using key clinical data. This analysis allowed us to examine associations between *S. pneumoniae* and clinical comorbidities, such COPD and CAD, as well as smoking, which were more prevalent in CAP patients than in healthy controls (Table 1). By integrating these variables, we aimed to assess their relationship with microbiome composition and determine how these comorbidities correlate with the presence of *S. pneumoniae*, potentially influencing disease progression.

Given that viral infections, such as influenza A can be exacerbated with a pneumococcal superinfection with an increased risk of severe disease progression, we included the results of viral tests in our model. Additionally, other relevant clinical variables, such as length of hospital stay (LOS), contact with children, disease severity (measured by the pneumonia severity index [PSI] score), and the presence of invasive disease (indicated by *S. pneumoniae* -positive blood culture results), were also considered in this analysis (Table S9).

**Fig. 5:**
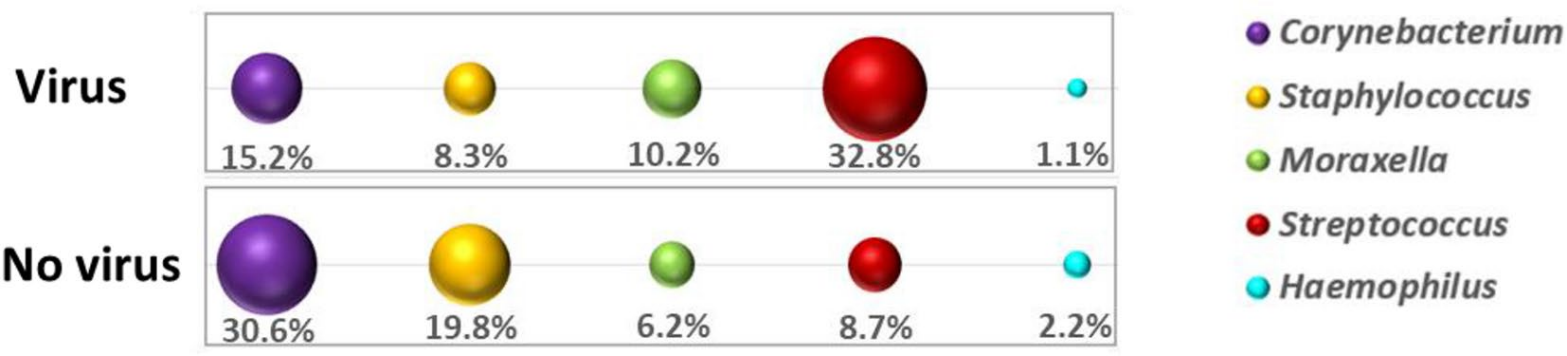
Bacterial taxonomic profiling of patients´ nasopharyngeal swabs collected during infection and grouped based on the presence or absence of a viral co-infection

Of all variables analyzed, only viral co-infection was found to be significantly associated (*p* = 0.001) with the presence of *Streptococcus* spp. in the nasopharynx. Notably, we did not find any correlation between the relative abundance of *Streptococcus* spp. in the nasopharynx and the occurrence of invasive disease. Further analysis of bacterial taxonomic communities revealed a higher relative abundance of *Streptococcus* spp. (mean RA 32.8%) in patients with viral co-infections compared to those without (mean RA 8.7%) (Fig. 5). In contrast, *Corynebacterium* spp. was the dominant taxon in patients without viral co-infection (mean RA 30.6%), whereas its mean relative abundance was lower in those with positive viral tests (mean RA 15.2%).

## Discussion

This study investigated the dynamics of the nasopharyngeal microbiome in 61 confirmed *S. pneumoniae*-infected adult patients both at admission to the hospital and three months post-infection. Findings in patients with *S. pneumoniae* CAP were compared to those obtained from 61 healthy control subjects. During infection, bacterial biomass analysis showed no significant differences between patients and controls; however, microbiome sequencing revealed significant differences in microbiome diversity. Notably, variability in bacterial biomass among controls was driven by a few outliers, while the overall sample remained homogeneous. Similar interindividual variance has been reported in previous studies, suggesting it is an inherent feature of the healthy microbiome [21, 22]. In contrast to the other groups, post-infection samples showed lower bacterial biomass and higher alpha diversity. Moreover, calculations of the modularity index in network analysis indicated that patients post-infection had lower microbiome resilience compared to samples obtained from either infected patients or healthy controls. Taxonomically, while both cohorts shared similar microbiomes, *Streptococcus* spp. was more abundant during the infection phase among pneumonia patients. Unique and significant bacterial interactions emerged during and after infection, differing from those observed in healthy individuals. Importantly, a significant negative correlation between *Corynebacterium* and *Streptococcus* spp. was identified, with the dominance of *Streptococcus* spp. linked to a reduced relative abundance of *Corynebacterium* spp. This antagonistic relationship between *Corynebacterium* and *Streptococcus* spp. is recently well-documented and suggests that *Corynebacterium* may play a protective role by inhibiting pathogen colonization [10]. Additionally, viral co-infections correlated with increased levels of *Streptococcus* spp., highlighting the impact of respiratory viruses on microbial dynamics [23]. No significant links were found between invasive disease, CAD or COPD and microbiome composition.

The detection of *S. pneumoniae* in this study was confirmed using a combination of diagnostic methods, as no single test achieved more than 69% positive detection. The urinary antigen detection (UAD) test had the highest detection rate at 69% followed by *S. pneumoniae* -specific PCR (58%). To confirm all cases, multiple diagnostic tests were necessary. Additionally, *16 S rRNA* gene amplicon sequencing detected *Streptococcus* spp. in 98.5% of patient samples, although results showed high variability across samples. These findings highlight the challenges in establishing a gold standard diagnostic method for pneumococcal pneumonia in adults.

In line with prior research [24], our results revealed only minor changes in the most dominant microbial communities between pneumococcal pneumonia patients and healthy controls, suggesting that the nasopharyngeal microbiota maintains a degree of stability and resilience, even during acute infection. Alpha diversity remained consistent between infected patients and healthy controls, but beta diversity showed significant differences, likely driven by the higher relative abundance of *Streptococcus* spp. found in the nasopharynx of patient samples during infection.

We observed a significant increase in α-diversity in samples taken three months post-infection, suggesting prolonged perturbations in the nasopharyngeal microbiome even after the resolution of acute illness. Interestingly Haak et al. [24]. observed no increase in microbial diversity within a one-month period, suggesting that the restructuring of the nasopharyngeal microbiome is a gradual process that occurs over a longer time frame. We next assessed microbiome stability through network analysis and modularity index calculations for each sample group, revealing a decline in modularity in post-infection samples, while the healthy microbiome exhibited the highest modularity index. The decreased modularity post-infection samples indicates lower microbiome resilience and incomplete recovery three months after pneumococcal CAP infection, likely influenced by antibiotic treatment and microbial clearance. Similarly, another study on the nasal cavity microbiome found that microbial communities may take several months to recover after antibiotic intervention [25]. Due to the lack of longitudinal data in the healthy control group, it remains unclear whether the microbiome in healthy individuals naturally fluctuates over time or remains stable due to their health status.

We observed a similar taxonomic profile between pneumococcal pneumonia patients and healthy individuals, with a core microbiome consisting of five main bacterial taxa: *Corynebacterium* spp., *Staphylococcus* spp., and either *Streptococcus* spp. (during the infection phase) or *Moraxella* spp. (in post-infection and healthy samples), and *Dolosigranulum* spp. as similarly shown elsewhere [26]. However, a high degree of variability in microbiome composition was observed across all samples, potentially influenced by factors such as age, smoking, and antibiotic use, as already reported by other studies [5, 27–30].

During infection, some patients showed a dominance of *Streptococcus* spp., while others had *Corynebacterium* spp. as the most abundant taxa. Our correlation analysis revealed a significant negative relationship between these two bacterial taxa, consistent with previous studies suggesting that *Corynebacterium* spp. may inhibit *Strept. pneumoniae* growth. For instance, this interaction has been observed in the nasopharyngeal microbiome of young children, in vitro, and in mouse models [31–33]. Similarly, significant correlations between *Corynebacterium* and *Staphylococcus* spp. were noted in nasal microbiome studies [34, 35], highlighting *Corynebacterium’s* role in microbial balance and its potential as a probiotic to enhance pathogen resistance in the nasopharynx, as already suggested elsewhere [36].

In post-infection samples, associations between *Prevotella* and *Veillonella* spp. were observed, two genera previously identified as potential commensals contributing to lung microbiome homeostasis [37]. The positive correlations between these genera suggest synergistic relationships that may promote microbiome stability, potentially offering colonization resistance against pathogens, particularly during recovery after antibiotic treatment.

To better understand the microbial interplay during infection with *S. pneumoniae*, post-infection and in healthy states, we conducted a targeted literature search focused on the significant bacterial interactions identified in this study. Interesting, we found limited information on bacterial associations within the nasopharyngeal microbiome, particularly in adults. Most research focuses on children [35, 38–40], leaving a gap in our understanding of bacterial interactions during and after infection in the adult population. This gap highlights the importance of investigating bacterial interplay in adult respiratory tracts, particularly for its potential roles in pathogen colonization resistance and disease progression in both upper and lower respiratory tracts. These findings underscore the need for further research into nasopharyngeal microbial interactions, which could offer valuable insights into *S. pneumoniae* colonization, pneumonia development, and the potential for microbiome-targeted therapies for respiratory infections.

Our findings confirmed the co-presence of respiratory viruses and *Streptococcus* spp. in the nasopharynx during infection consistent with previous studies [23, 41]. We identified a significant positive correlation between the relative abundance of *Streptococcus* spp. and viral co-infections, supporting the well-established notion that viral infections facilitate *S. pneumoniae* colonization by disrupting mucosal barriers and modulating immune responses [23]. The complex interplay between viruses and bacteria in the nasopharyngeal microbiome warrants further investigation, particularly in understanding its influence on microbiome disruption and how these factors might affect disease progression and outcomes.

Our study has several limitations. First, the relatively small sample size and the lack of direct comparisons with other respiratory tract sites, such as the lungs, limit our understanding of the relationship between the nasopharyngeal and lower respiratory tract microbiomes. Second, in nasopharyngeal swabs used for culture, only selected bacteria with pathogenic potential were considered, restricting direct comparisons between culture results and bacterial microbiome data obtained through sequencing. Additionally, the absence of follow-up sampling in the control group limits insights into microbiome fluctuations over time, particularly regarding microbiome stability. Moreover, a baseline sample collected before infection would have been ideal for better estimating the healthy composition of the microbiome in these patients and assessing its recovery after infection. The small sample size also hinders a detailed assessment of microbiome composition differences associated with specific *S. pneumoniae* serotypes. Given the varying prevalence of different serotypes in invasive disease versus carriage, this remains an important area for further investigation [9]. Furthermore, sequencing methods with higher resolution than V4 region amplification of the *rRNA* gene, such as shotgun metagenomics, could have improved the classification of *Streptococcus* spp. sequences at the species level. Absolute quantification and functional analysis would have further enhanced our understanding of in microbiome dynamics during and after infection, as well as microbial interactions in health and disease. Future studies should address these limitations to provide a more comprehensive perspective on the microbiome’s role in upper respiratory tract infections.

In conclusion, this study shows that *16 S rRNA* gene amplicon sequencing was the only method that detected *Streptococcus* spp. in nearly all samples compared to the other diagnostic tests performed for routine detection of *S. pneumoniae* in CAP patients. Additionally, our study demonstrates that adult patients with pneumococcal pneumonia experience ongoing disruption of the nasopharyngeal microbiome, characterized by reduced resilience and significant changes in microbial interactions three months after infection. We also identified unique bacterial interactions during and after infection that differ from those observed in healthy individuals. These microbial dynamics may be crucial for resistance to pathogen colonization. Overall, these results underscore the need for further investigation into microbial interactions and the potential for developing microbiome-targeted therapies to address respiratory infections, especially in at-risk populations.

## Electronic supplementary material

Below is the link to the electronic supplementary material.

Supplementary Material 1

## Data Availability

The data generated in this study are accessible in the SRA online repository under the Bioproject number: PRJNA1172557.
