# Supplementary material for: Profiling the nasopharyngeal Microbiome in patients with community-acquired pneumonia caused by Streptococcus pneumoniae: diagnostic challenges and ecological insights
Source: Med Microbiol Immunol. 2025 Apr 10;214(1):19. doi: 10.1007/s00430-025-00828-0 (PMC11985632; doi:10.1007/s00430-025-00828-0)
Supplement: Supplementary file 1 — Supplementary Material 1 [file 430_2025_828_MOESM1_ESM.docx]

**Supplementary material**

**Profiling the Nasopharyngeal Microbiome in Patients with Community-Acquired Pneumonia Caused by *Streptococcus pneumoniae*: Diagnostic Challenges and Ecological Insights**

Cristina Zubiria-Barrera^1,2,3^*, Linda Yamba Yamba^4,6^*, Tilman E. Klassert^1,2,3^, Malena Bos^1,2,3^, Jonas Ahl^4,5^, Lisa Wasserstrom^4,6^, Hortense Slevogt^1,2,3¶^ and Kristian Riesbeck^4,6 ¶^

* These two authors share the first authorship

^¶^ These two senior authors share the last authorship

*^1^Department of Respiratory Medicine and Infectious Diseases, MHH, German Center for Lung Research (DZL), BREATH, Hannover*

*^2^Respiratory Infection Dynamics Group, Helmholtz Centre for Infection Research, Braunschweig*

*^3^Cluster of Excellence RESIST (EXC 2155), Hannover Medical School, Carl-Neuberg-Straße 1, 30625 Hannover, Germany*

*^4^Clinical Microbiology, Department of Translational Medicine, Faculty of Medicine, Lund University, Malmö, Sweden*

*^5^Infectious Diseases, Department of Translational Medicine, Faculty of Medicine, Lund University, Malmö, Sweden*

*^6^Clinical Microbiology, Infection Control and Prevention, Skåne University Hospital, Lund, Sweden*

**Material and Methods**

**Patient and controls from the ECAPS cohort**

The ECAPS cohort has included 518 patients and 493 seasonally matched asymptomatic controls between September 2016-September 2018 at Malmö University hospital in Skåne (Sweden) (1, 2). Inclusion criteria for patients were two or more out of ten predefined symptoms of pneumonia combined with a radiographic finding. The healthy controls consisted of asymptomatic patients admitted to the Department of Orthopedics during the same period. Patients and controls were sampled with a nasopharyngeal swab at inclusion to the study within 48 hours of admission for the microbiome analyses and qPCR of selected viruses/bacteria. Patients were also invited to a follow up visit 10-17 weeks after discharge, where a second nasopharyngeal swab sample post-infection was collected to suffice a comparative analysis of the microbiome post-infection. Patients or controls with known immunodeficiency (Including AIDS/HIV or immunosuppressive therapy) or organ transplantation in the cohort were excluded for the microbiome analysis **Figure S1**. Healthy Controls included in the study were matched by sex, season, and age to patients included.

**Bacterial cultures at Clinical Microbiology, Laboratory Medicine Skåne**

Patient nasal swabs, blood and in some cases samples from the lower respiratory tract were used for microbial culture based on clinical indication by the treating clinician at admission. All included controls provided a nasopharyngeal swab for culture. During the study culture results were collected from the patients’ medical charts after admission. Blood and respiratory tract samples were cultured according to standard methods at Clinical Microbiology Laboratory Medicine Skåne (3, 4).

Blood cultures were performed using the automatic BACTEC system (BD diagnostic systems, Sparks, MD). Upon positivity in the BACTEC system Gram staining and species identification using the Maldi Sepsityper Kit® (Bruker Daltonics, Bremen, Germany) was performed. Positive blood cultures were then recultured on agar plates in CO_2_ 35-37°C (Blood and Gc-D) and aerobic 35-37°C (UriSelect) conditions for one day and in 35-37°C anaerobic (fastidious anaerobic with gentamicin disc) conditions for two days. Based on Gram staining and MALDI Biotyper analysis (Bruker Daltonics, Bremen, Germany) specific agar plates (Staph aureus Chromagar, Cephalexin-aztreonam-arabinose, Palcam, Candida chrom, Sabourad dextrose with kanamycin) were added for species identification the following day if indicated.

Lower respiratory tract samples were cultured on agar plates at 35-37°C in aerobic (UriSelect with vancomycin), anaerobic (P-BloodX with optochin disc) and CO_2_ conditions (Hematin with colistin disc, Columbia nalidixic acid agar with optochin disc and Hematin with bacitracin). Nasopharyngeal samples were cultured at 35-37°C in CO_2_ conditions (Hematin and Columbia Nalidixic Acid agar with optochin disc) for a total of two days.

For species identification following culture, colony morphology on above mentioned plates together with standard biochemical tests (optochin-sensitivity, oxidase, catalase and indole test) and MALDI Biotyper analysis was used. Formic acid was used when the laboratory technician deemed it useful for extraction and during the study period four different MALDI libraries were used by the Clinical Microbiology lab (MBT Compass Library DB-5989, MBT Compass Library DB-6903, MBT Compass Library DB-7311 and MBT Compass Library DB-7854). Coagulase-negative staphylococci were regarded as contaminants in blood cultures. Reported bacteria and fungi in respiratory tract cultures are focused on clinically relevant pathogens, while commensal flora with low pathogenic potential is generally not included in routine reporting. Minimum criteria for species identification of *Streptococcus pneumoniae* was defined as alpha-hemolysis on blood agar together with optochin sensitivity, for *Haemophilus influenzae* identification through MALDI Biotyper analysis and for *Moraxella catarrhalis* MALDI Biotyper analysis or typical appearance with solid colonies and oxidase positivity.

**Serotyping**

Serotyping was performed on available pneumococcal isolates using multiplex PCR and/or Immulex™ Pneumotest Kit (Statens Seruminstitut, Copenhagen, Denmark) according to instructions followed by Neufeldt Antisera (Statens Seruminstitut, Copenhagen, Denmark) as described by Uddén *et al.* or documented from UAD results (5).

**BinaxNOW S. pneumoniae^®^ / Urine antigen detection (UAD) and Legionella Urine antigen test**

Urine testing BinaxNOW *S. pneumoniae*® detects pneumococcal polysaccharide antigens in the urine. The UAD tests UAD1 and UAD2 detects 24 pneumococcal serotype-specific polysaccharides (serotypes 1, 3, 5, 6A, 6B, 7F, 9V, 14, 18C, 19A, 19F 23F, 2, 8, 9N, 10A, 11A, 12F, 15B/C, 17F, 20, 22F and 33F). Urine was collected upon inclusion to the study and urine antigen testing as well as the UAD serotype specific urine antigen detection assay was performed on all patients and some of the controls (6-8). Data was collected from the charts regarding Legionella urine antigen tests that were analyzed based on clinical indication in 32/61 included patients and no controls. Two different tests were used during the study period by the Clinical Microbiology lab in, Alere BinaxNOW and later ImmuView that both detect serogroup 1 antigens of *Legionella pneumophila*.

**Bacterial and viral detection by real-time PCR**

Available patients and 241 controls were also tested using PCR for detection of respiratory pathogens in the upper respiratory tract in the ECAPS cohort to establish different etiologies of pneumonia (1). Most patients in the microbiome study 60/61, were subjected to the below mentioned analysis on the same original sample as the microbiome analyses was performed. Only some of the included healthy controls 34/61 had available results.

The nasopharyngeal samples were stored at -80 ºC prior to real-time PCR analysis. MagNA Pure 96 DNA was used for extraction of DNA/RNA from 200 µl of the nasopharyngeal sample without pretreatment. For this the Viral NA Small volume kit (Roche Diagnostics, Basel, Switzerland) with addition of 20 µl poly(A) (Roche Diagnostics, Basel, Switzerland) was used per sample. RT Realtime-PCR amplifications for viral agents were performed on an ABI 7500 real-time PCR system (Applied Biosystems, Waltham, USA) in five multiplex reactions (9, 10). The reactions detect influenza A (H1N1/H3N2), influenza B, respiratory syncytical virus (RSV) A/B, human metapneumovirus (hMPV), parainfluenza virus (PIV) 1 to 3, adenovirus, coronavirus (OC43, NL63, 229E) and rhino-/enterovirus and parechovirus. For all reactions Path-ID Multiplex One-Step RT-PCR Kit (Ambion, Life Technologies, Carlsbad, USA) was used. The PCR program used for all viral reactions were a reverse transcription step at 48 ºC for 10 minutes and a RT-inactivation and initial denaturation step at 95 ºC for 10 minutes followed by 45 cycles of PCR-amplification at 95 ºC for 15 seconds and 55 ºC for 45 seconds.

Real-time PCR amplification for bacterial detection were performed using SensiFAST Probe No-ROX Kit (Bioline, Meridian Bioscience, Cincinnati, USA) and a Bio-Rad CFX96 (Bio-Rad, Hercules, USA). Detection of *Haemophilus influenzae* (11) and *Streptococcus pneumoniae* was performed. Detection of *lytA* in Streptococcus pneumoniae was an in house method developed at the Clinical Microbiology Department (Lund) using forward primer LytA-1-F 5’- GCTGGGTCAAGTACAAGGACACT-3, reverse primer LytA-1-R 5’- GTCCGCTGACTGGATAAAGGCA-3’ and probe Lyt-P 5’- FAM- ACTTAGACGCTAAAGAAGGCGCCATGGTATC-BHQ-1-3’.The PCR program used for *H. influenzae* and *S. pneumoniae* was a denaturation step at 95 ºC for 3 minutes followed by 45 cycles of PCR-amplification at 95 ºC for 10 seconds and 60 ºC for 50 seconds.

Also, atypical bacteria were tested, *M. pneumoniae*, *C. pneumoniae*, *B. pertussis* and *B. parapertussis* using a denaturation step at 95 ºC for 5 minutes followed by 45 cycles of PCR-amplification at 95 ºC for 10 seconds and 60 ºC for 30 seconds (12-14).

**Table S1: Primer constructs for Illumina *16S rRNA* amplicon sequencing**

| **Forward construct** | **Components** | **Sequence** |
| --- | --- | --- |
|  | 5’ - Illumina Adapter | AATGATACGGCGACCACCGAGATCTACACGCT |
|  | Golay barcode | NNNNNNNNNNNN |
|  | Forward primer pad | TATGGTAATT |
|  | Forward linker | GG |
|  | Forward primer (F515) | GTGYCAGCMGCCGCGGTAA |
| **Reverse construct** | **Components** | **Sequence** |
|  | 3’ - Illumina Adapter | CAAGCAGAAGACGGCATACGAGAT |
|  | Reverse primer pad | AGTCAGCCAG |
|  | Reverse linker | CC |
|  | Reverse primer (R806) | GGACTACNVGGGTWTCTAAT |

**
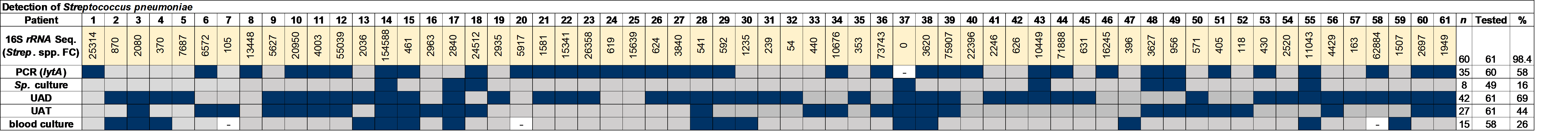
Table S2. Summary of diagnostic tests performed on nasopharyngeal swab samples from pneumonia patients during infection.**

*Strep. spp*. = *Streptococcus* spp.; Sp. = *Streptococcus pneumoniae*; FC = feature counts; UAD = Urine antigen detection; UAT = Urine antigen test

dark blue = positive test; grey = negative test;

- = no test performed

**Table S3. Serotype distribution of detected serotypes.** Among the included patient 45/61 had one or several serotypes detected using urine antigen detection and serotyping of isolates from nasopharyngeal culture and blood culture. Four individuals had two different serotypes detected.

| **Serotype** | **3** | **8** | **9N** | **19A** | **4** | **11A** | **22F** | **5** | **33F** | **14** | **37** | **10A** | **17F** | **18C** | **15B** | **19F** | **23F** | **35B** | **6A** | **12F** |
| --- | --- | --- | --- | --- | --- | --- | --- | --- | --- | --- | --- | --- | --- | --- | --- | --- | --- | --- | --- | --- |
| % | 27 | 11 | 11 | 9 | 7 | 7 | 4 | 4 | 4 | 2 | 2 | 2 | 2 | 2 | 2 | 2 | 2 | 2 | 2 | 2 |
| *n* | 12 | 5 | 5 | 4 | 3 | 3 | 2 | 2 | 2 | 1 | 1 | 1 | 1 | 1 | 1 | 1 | 1 | 1 | 1 | 1 |

**Table S4: Feature counts of nasopharynx core microbiome**

| **Patients** |  | **Healthy individuals** |
| --- | --- | --- |
| **Infection** | **Post-infection** |  |
| *Corynebacterium* spp. (mean 22521 FC) | *Corynebacterium* spp. (mean 15618 FC) | *Corynebacterium* spp. (mean 20798 FC) |
| *Staphylococcus* spp. (mean 13795 FC) | *Staphylococcus* spp. (mean 10232 FC) | *Staphylococcus* spp. (mean 14308 FC) |
| *Streptococcus* spp. (mean 12916 FC) | *Moraxella* spp*.* (mean 5592 FC) | *Moraxella* spp*.* (mean 11090 FC) |
| *Moraxella* spp*.* (mean 6902 FC) | *Dolosigranulum* spp*.* (mean 5327 FC) | *Dolosigranulum* spp*.* (mean 10074 FC) |
| *Dolosigranulum* spp*.* (mean 4232 FC) | *Haemophilus* spp. (mean 3592 FC) | *Prevotella* spp. (mean 3461 FC) |

Shown are the top 5 main bacterial taxa for each group, ordered by mean feature counts (FC) detected

**Table S5: Differentially Abundant Bacterial Taxa between Patient Sampling Time Points and Healthy Controls measured by ANCOMBC**

| **Group 1 vs. Group 2** | **Taxa** | ***p-*value** | **FC_Group 1** | **FC_Group 2** | **RA %_Group1** | **RA %_Group2** |
| --- | --- | --- | --- | --- | --- | --- |
| **Infection vs. Post-infection** | *Streptococcus* spp*.* | 0.00004 | 12916.3 | 2011.4 | 14.6 | 3.9 |
|  | *Bacillus* spp*.* | 0.023 | 1987.8 | 1037.6 | 2.4 | 0.28 |
|  | *Paraclostridium* spp*.* | 0.032 | 644.1 | 106.5 | 0.80 | 0.14 |
| **Infection vs. Healthy Controls** | *Streptococcus* spp*.* | 0.00791 | 12916.3 | 1603.8 | 14.6% | 2.4% |

FC = mean feature counts; RA = mean relative abundance

**Table S6: Pearson correlation analysis between the relative abundance (RA) of *Streptococcus* spp. in nasopharynx samples of infected CAP patients and clinical diagnostic test results.**

|  | **Correlations** |  |  |
| --- | --- | --- | --- |
|  |  |  | ***Streptococcus* RA** |
| **Pearson** | **PCR** | Correlation coefficient  Sig. (2-tailed) | .413  .001** |
|  | **UAD** | Correlation coefficient  Sig. (2-tailed) | -.026  .841 |
|  | **UAT** | Correlation coefficient  Sig. (2-tailed) | .113  .385 |
|  | **Sp. positive blood culture** | Correlation coefficient  Sig. (2-tailed) | -.109  .417 |
|  | **NP** | Correlation coefficient  Sig. (2-tailed) | .139  .284 |

** Correlation is significant at the 0.01 level (2-tailed).

*n* = 61

**Table S7: Pearson correlation coefficients and *p*-values for the associations among bacterial taxa identified in nasopharyngeal samples**

| **Sampling** | **Significant bacterial associations**  **(genus level)** | **Pearson correlation coefficient (PCC)** | ***p*-value** |
| --- | --- | --- | --- |
| **Infection (I)** | *Corynebacterium - Staphylococcus* | -0.27 | 0.0325 |
|  | *Streptococcus - Corynebacterium* | -0.29 | 0.0249 |
|  | *Staphylococcus - Lawsonella* | 0.26 | 0.0408 |
|  | *Bacillaceae - Anaerococcus* | 0.36 | 0.00465 |
|  | *Peptoniphilus - Lawsonella* | 0.37 | 0.00356 |
|  | *Peptoniphilus - Anaerococcus* | 0.41 | 0.00119 |
|  | *Bacillaceae - Anoxybacillus.* | 0.61 | 1.49E-07 |
|  |  |  |  |
| **Post-infection (PI)** | *Corynebacterium - Prevotella* | -0.26 | 0.0437 |
|  | *Corynebacterium - Bacillaceae* | -0.31 | 0.0141 |
|  | *Anaerococcus - Prevotella* | 0.26 | 0.0417 |
|  | *Streptococcus - Veillonella* | 0.31 | 0.0147 |
|  | *Streptococcus - Prevotella* | 0.41 | 0.00116 |
|  | *Veillonella - Prevotella* | 0.61 | 0.000000157 |
|  | *Bacillaceae - Geobacillus* | 0.68 | 2.18E-09 |
|  | *Anaerococcus - Peptoniphilus* | 0.7 | 2.77E-10 |
|  |  |  |  |
| **Healthy controls (C)** | *Staphylococcus - Moraxella* | -0.27 | 0.0353 |
|  | *Staphylococcus - Dolosigranulum* | -0.28 | 0.0295 |
|  | *Corynebacterium - Staphylococcus* | -0.31 | 0.0165 |
|  | *Corynebacterium - Moraxella* | -0.34 | 0.00828 |
|  | *Veillonella - Neisseriaceae* | 0.34 | 0.00688 |
|  | *Bacillaceae - Veillonella* | 0.38 | 0.00235 |
|  | *Streptococcus - Bacillaceae* | 0.68 | 1.43E-09 |

blue: negative correlation; red: positive correlation

**Table S8: Literature review summarizing significant bacterial associations and correlations identified in the nasopharyngeal microbiome in this study.**

| **This study** |  |  | **Literature** |  |  |
| --- | --- | --- | --- | --- | --- |
| **Significant bacterial associations (genus level)** | **Study details** | **Study population** | **Children**  **/adults** | **Body site location** | **Source** |
| *Corynebacterium - Staphylococcus (I) / (C)* | *Corynebacterium pseudodiphtheriticum* inhibited *S. aureus* growth | healthy individuals | adults | nasal cavity | (15) |
|  | *Corynebacterium pseudodiphtheriticum* mediates contact-independent bactericidal activity against *S. aureus* | *in vitro (*clinical isolates) | - | nasal cavity | (16) |
|  | *Corynebacterium accolens* has antimicrobial activity against *S. aureus* | *in vitro (*clinical isolates) | adults | nasal cavity | (17) |
|  | Corynebacterium abundance negatively correlates with *S. aureus* | neonates in ICU (NICU) | infants | nasal cavity | (18) |
| *Streptococcus – Corynebacterium (I)* | *Corynebacterium* may protect against *Streptococcus pneumoniae* colonization in young children's nasopharyngeal microbiome | healthy children | children | nasopharynx | (19) |
|  | *Corynebacterium accolens* cultures in-  hibited *S. pneumoniae* growth through the lipase LipS1 | *in vitro* | - | - | (19) |
|  | *Corynebacterium* species Inhibit *Streptococcus pneumoniae* colonization and infection of the mouse airway | mice | - | lung | (20) |
|  | - | - | - | - | - |
| *Staphylococcus – Lawsonella (I)* | - | - | - | - | - |
| *Bacillaceae – Anaerococcus (I)* | - | - | - | - | - |
| *Bacillaceae – Anoxybacillus (I)* | - | - | - | - | - |
| *Peptoniphilus – Lawsonella (I)* | Co-ocurrence between *Anaerococcus* and *Peptoniphilus* in human chronic wounds | wound cotton swabs from adults | adults | skin | (21) |
| *Anaerococcus – Peptoniphilus (I) / (PI)* | - | - | - | - | - |

blue: negative correlation; red: positive correlation; (I): infection phase

| **This study** |  |  | **Literature** |  |  |
| --- | --- | --- | --- | --- | --- |
| **Significant bacterial associations**  **(genus level)** | **Study details** | **Study population** | **Children**  **/adults** | **Body site location** | **Source** |
| *Corynebacterium – Prevotella (PI)* | - | - | - | - | - |
| *Corynebacterium – Bacillaceae (PI)* | - | - | - | - | - |
| *Anaerococcus – Prevotella (PI)* | Co-ocurrence between *Anaerococcus* and *Prevotella* in human chronic wounds | wound cotton swabs from adults | adults | skin | (21) |
| *Streptococcus – Veillonella (PI)* | Veillonella. tobetsuensis produces signaling molecules that promote the proliferation of Streptococcus gordonii in biofilm formation | *in vitro* | - | oral cavity | (22) |
|  | Significant positive correlation between higher levels of Streptococcus mutans and Veillonella parvula/dispar. | root caries infection | elderly adults | oral cavity | (23) |
| *Streptococcus – Prevotella (PI)* | *Prevotella* species increased *S. pneumoniae* clearance, such as *P. melaninogenica*, activating neutrophils in a TLR2-dependent  manner | mice | - | lung | (24) |
| *Veillonella – Prevotella (PI)* | - | - | - | - | - |
| *Bacillaceae – Geobacillus (PI)* | - | - | - | - | - |

blue: negative correlation; red: positive correlation; (PI): post-infection phase

| **This study** |  |  | **Literature** |  |  |
| --- | --- | --- | --- | --- | --- |
| **Significant bacterial associations**  **(genus level)** | **Study details** | **Study population** | **Children**  **/adults** | **Body site location** | **Source** |
| *Staphylococcus – Moraxella (C)* | D. pigrum inhibited S. aureus in vitro. | *in vitro* | - | nasal cavity | (25) |
| *Staphylococcus – Dolosigranulum (C)* | Dolosigranulum pigrum and S. aureus are inversely correlated in adult nasal microbiota | healthy subjects | Infants-adults | nasal cavity | (26) |
| *Corynebacterium – Moraxella (C)* | Strong negative correlations between the *Corynebacterium* and *Moraxella* | infants with CF vs. matched healthy controls | infants | nasopharynx | (27) |
| *Veillonella – Neisseriaceae (C)* | Veillonella and Neisseria spp. showed ability to attach to oral surfaces. | healthy subjects | adults | oral cavity | (28) |
| *Bacillaceae – Veillonella (C)* | - | - | - | - | - |
| *Streptococcus – Bacillaceae (C)* | - | - | - | - | - |

blue: negative correlation; red: positive correlation; (C): Healthy Controls

**Table S9: Pearson correlation analysis of the relative abundance (RA) of *Streptococcus spp*. in nasopharyngeal samples from infected CAP patients with clinical data**

|  | **Correlations** |  |  |
| --- | --- | --- | --- |
|  |  |  | ***Streptococcus* RA** |
| **Pearson** | **COPD** | Correlation coefficient  Sig. (2-tailed)  N | .224  .083  61 |
|  | **Coronary artery disease** | Correlation coefficient  Sig. (2-tailed)  N | .140  .357  61 |
|  | **Smoker (previously/actually)** | Correlation coefficient  Sig. (2-tailed)  N | .119  .623  61 |
|  | **Antibiotic treatment (past 14 days)** | Correlation coefficient  Sig. (2-tailed)  N | -.181  .162  61 |
|  | **Sp. positive blood culture** | Correlation coefficient  Sig. (2-tailed)  N | -.109  .417  61 |
|  | **Viral co-infection** | Correlation coefficient  Sig. (2-tailed)  N | .411  .001**  60 |
|  | **PSI score** | Correlation coefficient  Sig. (2-tailed)  N | .024  .486  61 |
|  | **Shannon index** | Correlation coefficient  Sig. (2-tailed)  N | -.034  .793  61 |
|  | **Length of stay (LOS) at hospital** | Correlation coefficient  Sig. (2-tailed)  N | .059  .793  61 |
|  | **Children contact** | Correlation coefficient  Sig. (2-tailed)  N | -.048  .713  61 |

** Correlation is significant at the 0.01 level (2-tailed).

* Correlation is significant at the 0.05 level (2-tailed).

**Figure S1. Inclusion of CAP patients diagnosed with *Streptococcus pneumoniae* from the ECAPS cohort.**

**
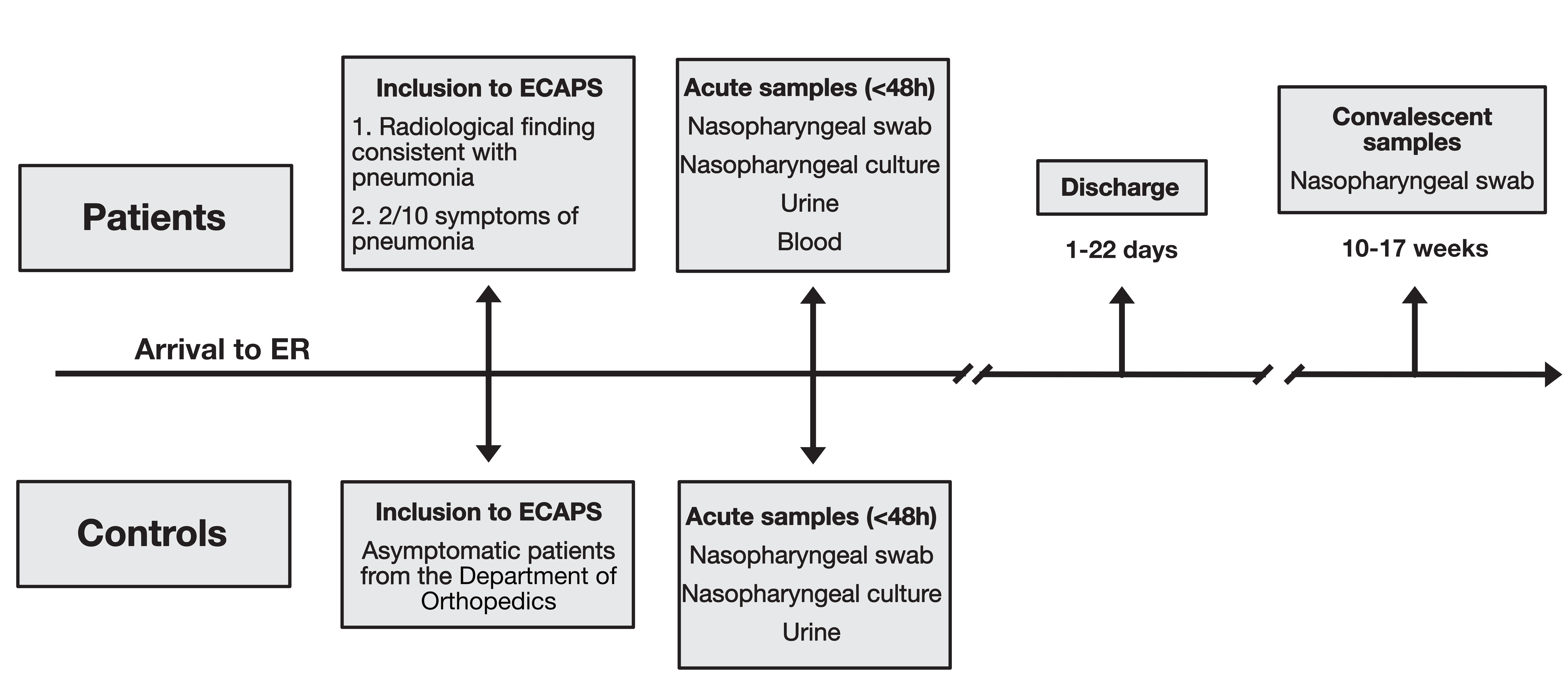
**

**Figure S2. Timeline of the sampling of patients and controls.** Patients were screened after arrival to the ER for inclusion to the ECAPS cohort. Diagnosis of CAP was defined as a radiographic finding indicating pneumonia and at least 2 out of 10 clinical symptoms of pneumonia. Controls with no respiratory or other infectious symptoms in the previous 14 days were included from the Department of Orthopedics. A nasopharyngeal sample was taken within 48 h of admission from both patients and controls to represent a community acquired microbiome. Patients were sampled a second time 10-17 weeks after discharge. **Abbreviations: ECAPS** - Etiology of community-acquired pneumonia in Sweden; **ER** -Emergency room.

**
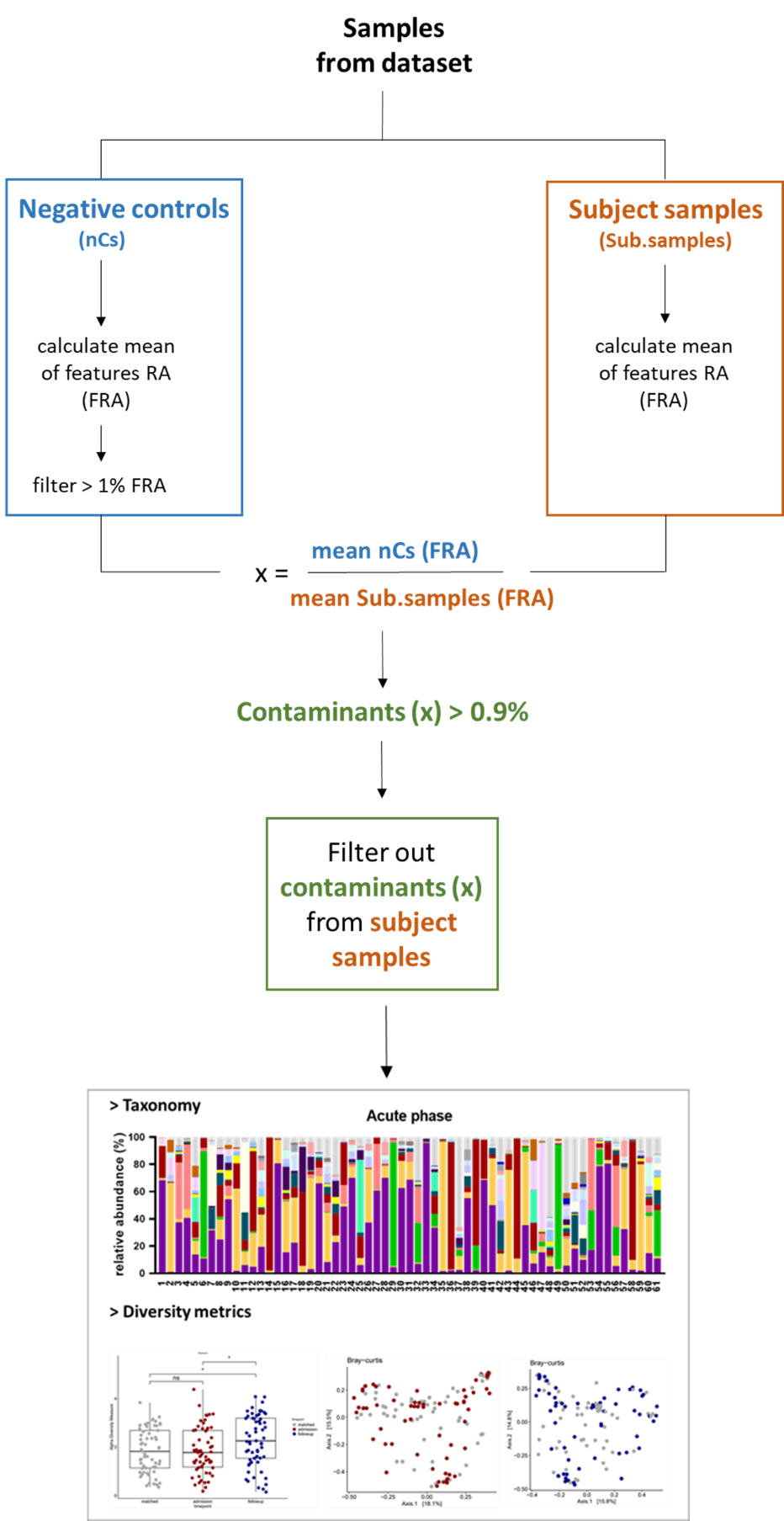
**

**Figure S3: Workflow of our custom-developed contaminant feature-filtering bioinformatics pipeline for *16S rRNA* amplicon sequencing data**: A custom bioinformatics pipeline was developed in R to eliminate contaminant features detected in negative control samples from patient samples. The dataset was divided into subject and negative control groups. Features with a mean relative abundance (FRA) of less than 1% found in negative controls samples were discarded. A ratio was calculated between the mean FRA in negative controls and individual samples. Features with a ratio greater than 0.9 were identified as contaminants and subsequently removed from the subject sample dataset.


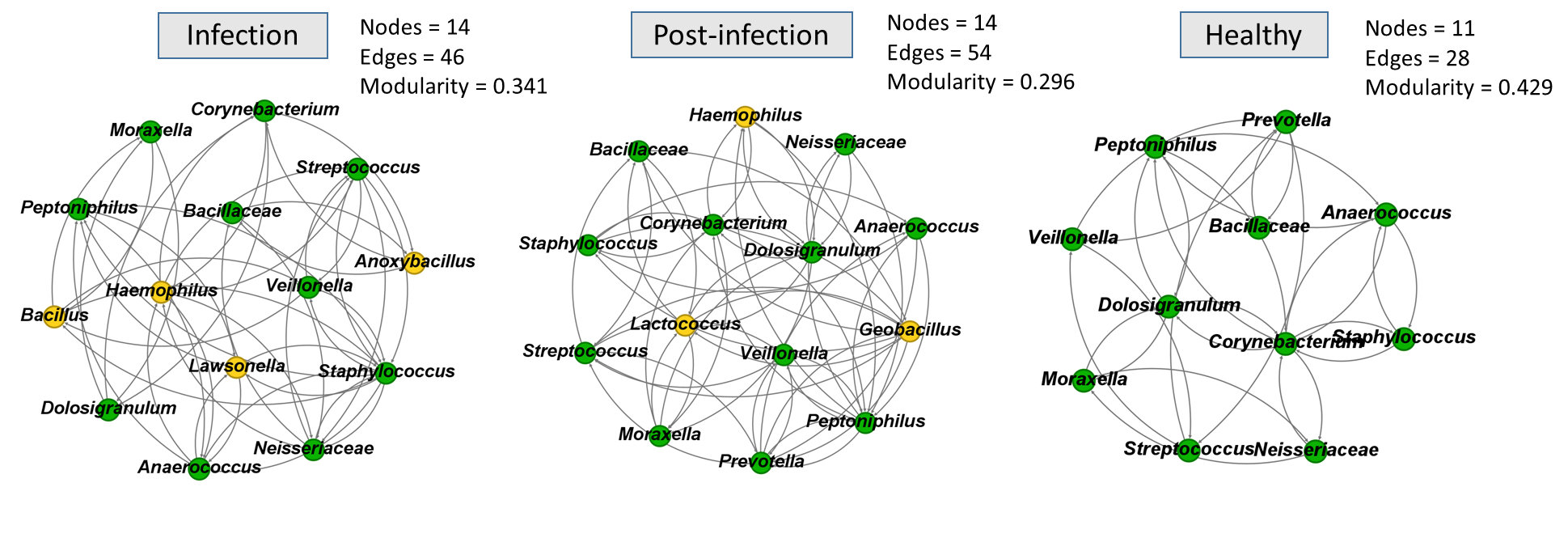

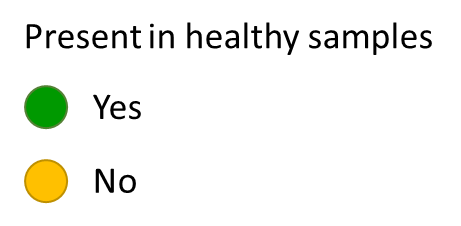


**Figure S4: Network analysis of correlation coefficients (calculated using SparCC) from nasopharyngeal microbiome samples**. The network diagram represents correlation coefficients greater than 0.1 for bacterial taxa with a mean relative abundance of over 1% in both patient and healthy control samples. Bacterial taxa are represented by nodes, colored green for taxa present in healthy samples, and yellow for those absent. The number of connections (edges), nodes, and the modularity index are indicated for each network.

**
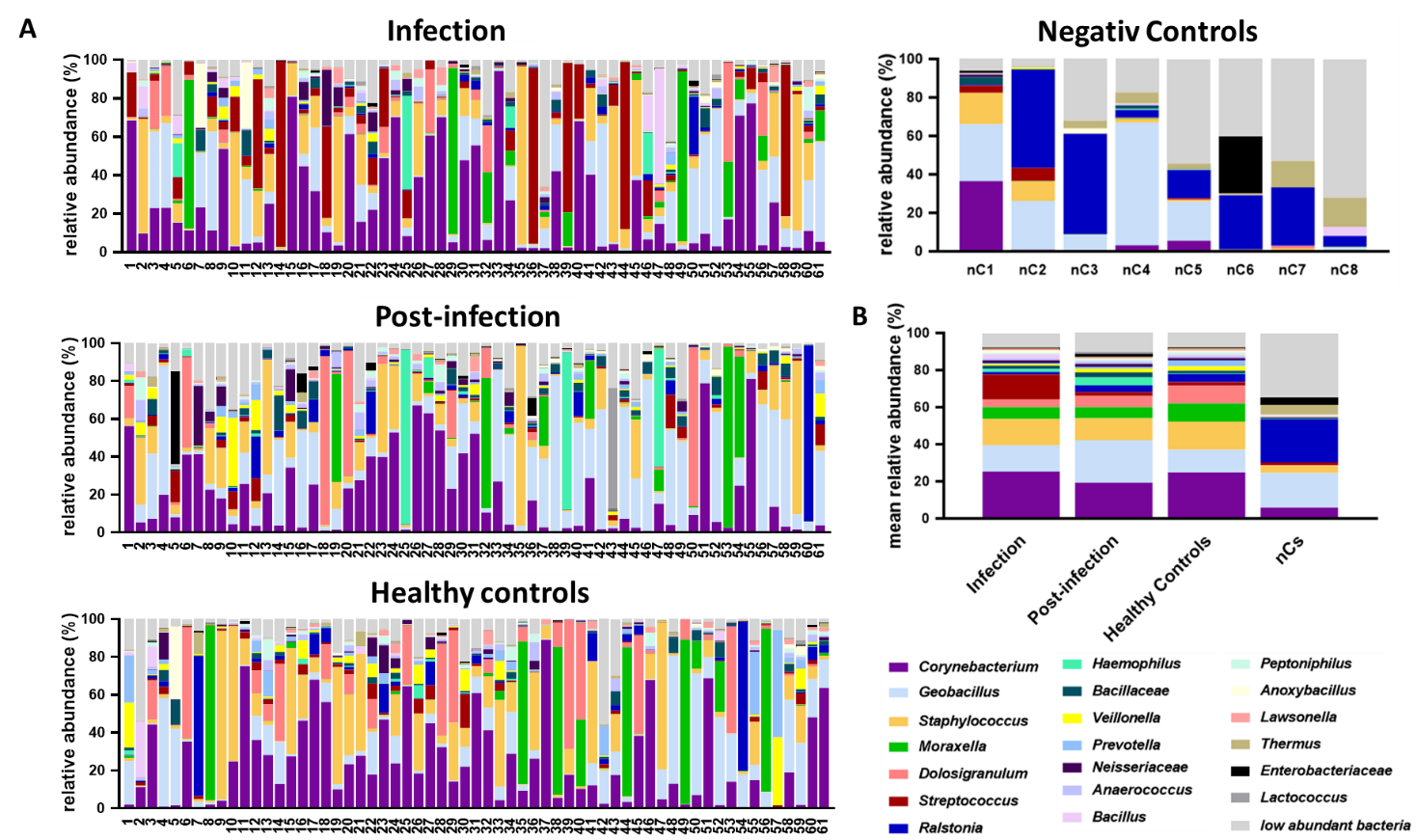
**

**Figure S5. Bacterial taxonomic composition of nasopharyngeal swab samples from CAP patients and wet-lab negative controls**. (A) Relative abundance of bacterial taxa at the genus level in CAP patients, matched control samples, and negative controls. (B) Mean relative abundance of bacterial taxa in each group analyzed.

**
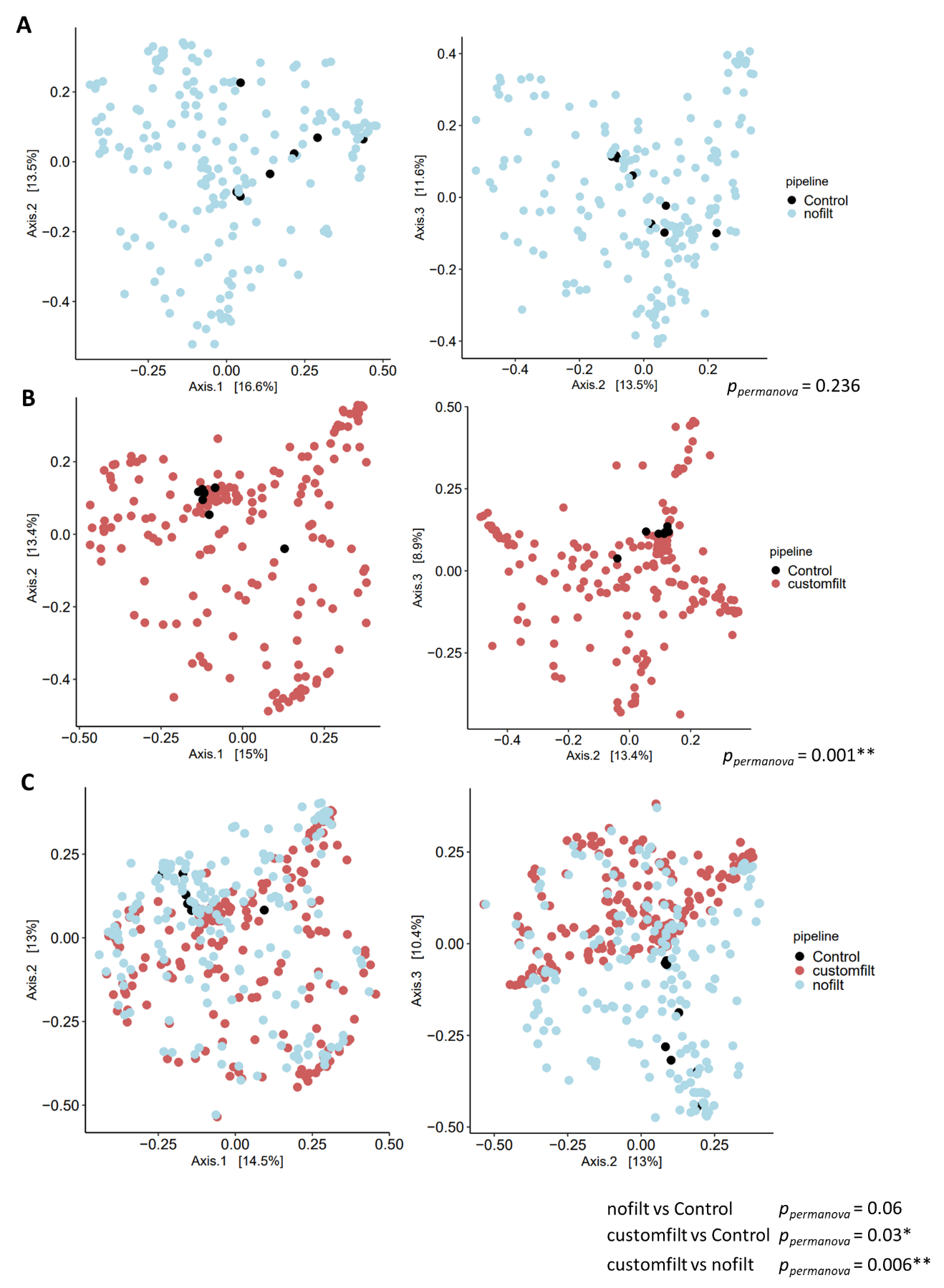
**

**Figure S6. Beta diversity analysis between samples and negative controls. Bioinformatics pipelines comparison. (A)** Principal coordinate analysis (PCoA) of the beta diversity using Bray-Curtis distances for samples and negative controls analyzed with a bioinformatics pipeline that does not include any contaminants filtering steps (nofilt) **(B)** Application of our custom bioinformatics feature-filtering pipeline on the dataset (customfilt). PCoA showing Bray-Curtis distances of negative controls and filtered samples  **(C)** PCoA of Bray-Curtis distances for samples with (customfilt) and without (nofilt) the application of our custom contaminant filtering bioinformatics pipeline, along with negative control samples. P-values correspond to pairwise comparisons using PERMANOVA. * Significant p-values (p < 0.05).


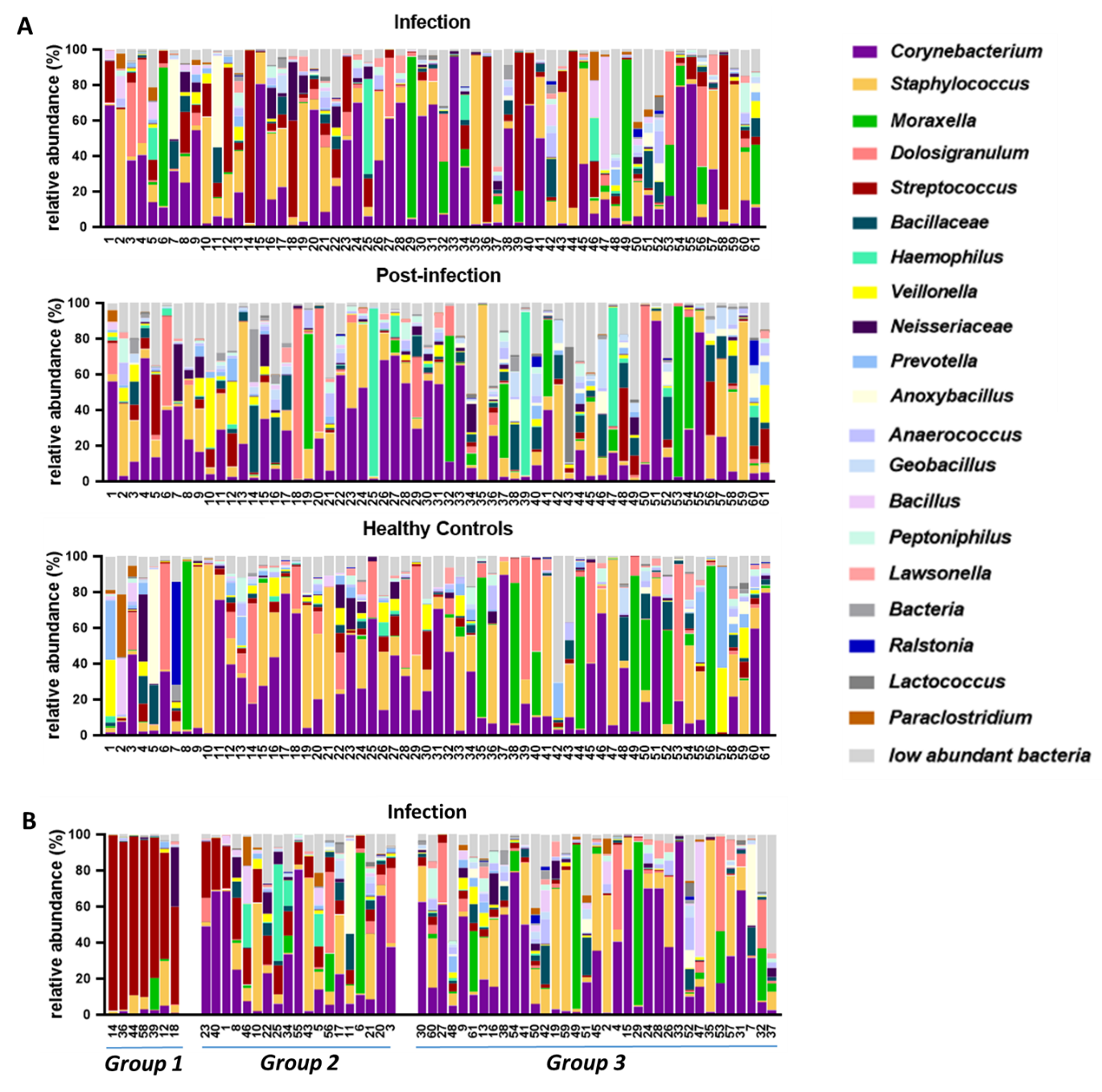


**Figure S7**: **Bacterial taxonomic composition of nasopharyngeal swabs samples from pneumococcal CAP patients and healthy individuals**. **(A)** Taxonomic bacterial communities in nasopharyngeal swab samples from *S. pneumoniae* CAP patients during infection and post-infection phases, compared to samples from healthy individuals. The 20 most abundant genera are illustrated for each group. **(B)** Categorization of CAP samples from the infection phase based on the relative abundance of *Streptococcus* spp.: Group 1 (>50% RA), Group 2 (50%-5% RA), and Group 3 (<5% RA).

**References**

1. Hansen K, Yamba Yamba L, Wasserstrom L, Rünow E, Göransson T, Nilsson A, et al. Exploring the microbial landscape: uncovering the pathogens associated with community-acquired pneumonia in hospitalized patients. Frontiers in Public Health. 2023;11.

2. Hansen K, Rünow E, Torisson G, Theilacker C, Palmborg A, Pan K, et al. Radiographically confirmed community-acquired pneumonia in hospitalized adults due to pneumococcal vaccine serotypes in Sweden, 2016–2018—The ECAPS study. Frontiers in Public Health. 2023;11.

3. Jorgensen JH, Pfaller MA, Carroll KC, American Society for M. Manual of clinical microbiology. 11th edition ed. Washington, DC: ASM Press Washington, DC; 2015.

4. Claesson B, Hallander H, Nyberg A, Thore M, Wollin R, Schalén C, et al. Referensmetodik för laboratoriediagnostik vid kliniskt mikrobiologiska laboratorier. 2 ed: Föreningen för klinisk mikrobiologi; 2005.

5. Uddén F, Rünow E, Slotved HC, Fuursted K, Ahl J, Riesbeck K. Characterization of Streptococcus pneumoniae detected in clinical respiratory tract samples in southern Sweden 2 to 4 years after introduction of PCV13. J Infect. 2021;83(2):190-6.

6. Kalina WV, Souza V, Wu K, Giardina P, McKeen A, Jiang Q, et al. Qualification and Clinical Validation of an Immunodiagnostic Assay for Detecting 11 Additional Streptococcus pneumoniae Serotype-specific Polysaccharides in Human Urine. Clin Infect Dis. 2020;71(9):e430-e8.

7. Pride MW, Huijts SM, Wu K, Souza V, Passador S, Tinder C, et al. Validation of an immunodiagnostic assay for detection of 13 Streptococcus pneumoniae serotype-specific polysaccharides in human urine. Clin Vaccine Immunol. 2012;19(8):1131-41.

8. Hansen K, Rünow E, Torisson G, Theilacker C, Palmborg A, Pan K, et al. Radiographically confirmed community-acquired pneumonia in hospitalized adults due to pneumococcal vaccine serotypes in Sweden, 2016-2018-The ECAPS study. Front Public Health. 2023;11:1086648.

9. Ek P, Böttiger B, Dahlman D, Hansen KB, Nyman M, Nilsson AC. A combination of naso- and oropharyngeal swabs improves the diagnostic yield of respiratory viruses in adult emergency department patients. Infect Dis (Lond). 2019;51(4):241-8.

10. Østby AC, Gubbels S, Baake G, Nielsen LP, Riedel C, Arpi M. Respiratory virology and microbiology in intensive care units: a prospective cohort study. Apmis. 2013;121(11):1097-108.

11. Smith-Vaughan H, Byun R, Nadkarni M, Jacques NA, Hunter N, Halpin S, et al. Measuring nasal bacterial load and its association with otitis media. BMC Ear Nose Throat Disord. 2006;6:10.

12. Hardegger D, Nadal D, Bossart W, Altwegg M, Dutly F. Rapid detection of Mycoplasma pneumoniae in clinical samples by real-time PCR. J Microbiol Methods. 2000;41(1):45-51.

13. Welti M, Jaton K, Altwegg M, Sahli R, Wenger A, Bille J. Development of a multiplex real-time quantitative PCR assay to detect Chlamydia pneumoniae, Legionella pneumophila and Mycoplasma pneumoniae in respiratory tract secretions. Diagn Microbiol Infect Dis. 2003;45(2):85-95.

14. Roorda L, Buitenwerf J, Ossewaarde JM, van der Zee A. A real-time PCR assay with improved specificity for detection and discrimination of all clinically relevant Bordetella species by the presence and distribution of three Insertion Sequence elements. BMC Research Notes. 2011;4(1):11.

15. Yan M, Pamp SJ, Fukuyama J, Hwang PH, Cho D-Y, Holmes S, et al. Nasal microenvironments and interspecific interactions influence nasal microbiota complexity and S. aureus carriage. Cell host & microbe. 2013;14(6):631-40.

16. Hardy BL, Dickey SW, Plaut RD, Riggins DP, Stibitz S, Otto M, et al. Corynebacterium pseudodiphtheriticum exploits Staphylococcus aureus virulence components in a novel polymicrobial defense strategy. MBio. 2019;10(1):10.1128/mbio. 02491-18.

17. Menberu MA, Liu S, Cooksley C, Hayes AJ, Psaltis AJ, Wormald P-J, et al. Corynebacterium accolens has antimicrobial activity against Staphylococcus aureus and methicillin-resistant S. aureus pathogens isolated from the sinonasal niche of chronic rhinosinusitis patients. Pathogens. 2021;10(2):207.

18. Khamash DF, Mongodin EF, White JR, Voskertchian A, Hittle L, Colantuoni E, et al., editors. The association between the developing nasal microbiota of hospitalized neonates and Staphylococcus aureus colonization. Open forum infectious diseases; 2019: Oxford University Press US.

19. Bomar L, Brugger SD, Yost BH, Davies SS, Lemon KP. Corynebacterium accolens releases antipneumococcal free fatty acids from human nostril and skin surface triacylglycerols. MBio. 2016;7(1):10.1128/mbio. 01725-15.

20. Horn KJ, Jaberi Vivar AC, Arenas V, Andani S, Janoff EN, Clark SE. Corynebacterium species inhibit Streptococcus pneumoniae colonization and infection of the mouse airway. Frontiers in Microbiology. 2022;12:804935.

21. Choi Y, Banerjee A, McNish S, Couch KS, Torralba MG, Lucas S, et al. Co-occurrence of anaerobes in human chronic wounds. Microbial ecology. 2019;77:808-20.

22. Mashima I, Nakazawa F. Interaction between Streptococcus spp. and Veillonella tobetsuensis in the early stages of oral biofilm formation. Am Soc Microbiol; 2015.

23. Abram AM, Szewczyk MM, Park SG, Sam SS, Eldana HB, Koria FJ, et al. A co-association of Streptococcus mutans and Veillonella parvula/dispar in root caries patients and in vitro biofilms. Infection and immunity. 2022;90(10):e00355-22.

24. Horn KJ, Schopper MA, Drigot ZG, Clark SE. Airway Prevotella promote TLR2-dependent neutrophil activation and rapid clearance of Streptococcus pneumoniae from the lung. Nature Communications. 2022;13(1):3321.

25. Brugger SD, Eslami SM, Pettigrew MM, Escapa IF, Henke MT, Kong Y, et al. Dolosigranulum pigrum cooperation and competition in human nasal microbiota. Msphere. 2020;5(5):10.1128/msphere. 00852-20.

26. Accorsi EK, Franzosa EA, Hsu T, Joice Cordy R, Maayan-Metzger A, Jaber H, et al. Determinants of Staphylococcus aureus carriage in the developing infant nasal microbiome. Genome biology. 2020;21:1-24.

27. Prevaes SM, De Winter-De Groot KM, Janssens HM, de Steenhuijsen Piters WA, Tramper-Stranders GA, Wyllie AL, et al. Development of the nasopharyngeal microbiota in infants with cystic fibrosis. American journal of respiratory and critical care medicine. 2016;193(5):504-15.

28. Liljemark W, Gibbons R. Ability of Veillonella and Neisseria species to attach to oral surfaces and their proportions present indigenously. Infection and immunity. 1971;4(3):264-8.
